# Human cardiac ^31^P magnetic resonance spectroscopy at 7 tesla

**DOI:** 10.1002/mrm.24922

**Published:** 2013-09-04

**Authors:** Christopher T Rodgers, William T Clarke, Carl Snyder, J Thomas Vaughan, Stefan Neubauer, Matthew D Robson

**Affiliations:** 1 Oxford Centre for Clinical Magnetic Resonance Research (OCMR), University of OxfordLevel 0, John Radcliffe Hospital, Oxford, United Kingdom; 2 Center for Magnetic Resonance Research, University of Minnesota2021 Sixth Street SE, Minneapolis, Minnesota, USA.

**Keywords:** ^31^P magnetic resonance spectroscopy, phosphorus, cardiac, 7 Tesla, field strength, T_1_

## Abstract

**Purpose:**

Phosphorus magnetic resonance spectroscopy (^31^P-MRS) affords unique insight into cardiac energetics but has a low intrinsic signal-to-noise ratio (SNR) in humans. Theory predicts an increased ^31^P-MRS SNR at 7T, offering exciting possibilities to better investigate cardiac metabolism. We therefore compare the performance of human cardiac ^31^P-MRS at 7T to 3T, and measure T_1_s for ^31^P metabolites at 7T.

**Methods:**

Matched ^31^P-MRS data were acquired at 3T and 7T, on nine normal volunteers. A novel Look-Locker CSI acquisition and fitting approach was used to measure T_1_s on six normal volunteers.

**Results:**

T_1_s in the heart at 7T were: phosphocreatine (PCr) 3.05 ± 0.41s, γ-ATP 1.82 ± 0.09s, α-ATP 1.39 ± 0.09s, β-ATP 1.02 ± 0.17s and 2,3-DPG (2,3-diphosphoglycerate) 3.05 ± 0.41s (N = 6). In the field comparison (N = 9), PCr SNR increased 2.8× at 7T relative to 3T, the Cramer-Ráo uncertainty (CRLB) in PCr concentration decreased 2.4×, the mean CRLB in PCr/ATP decreased 2.7× and the PCr/ATP SD decreased 2×.

**Conclusion:**

Cardiac ^31^P-MRS at 7T has higher SNR and the spectra can be quantified more precisely than at 3T. Cardiac ^31^P T_1_s are shorter at 7T than at 3T. We predict that 7T will become the field strength of choice for cardiac ^31^P-MRS. Magn Reson Med 72:304–315, 2014. © 2013 The Authors. Magnetic Resonance in Medicine Published by Wiley Periodicals, Inc. on behalf of International Society of Medicine in Resonance. This is an open access article under the terms of the Creative Commons Attribution License, which permits use, distribution, and reproduction in any medium, provided the original work is properly cited.

## INTRODUCTION

Phosphorus magnetic resonance spectroscopy (^31^P-MRS) makes a unique and valuable contribution to our understanding of metabolism 1–4. Applied in the human heart 5, ^31^P-MRS reveals the biochemistry of ATP, ADP, and phosphocreatine (PCr), which are critical to the supply of energy for contractile work in the myocardium. For example, derangement of the ratio of concentrations of PCr to ATP measured by ^31^P-MRS (“the PCr/ATP ratio”) predicts mortality 2; diminution of the creatine-kinase flux is seen in patients with myocardial infarction 6; and changes in the PCr/ATP concentration ratio during pharmacological stress are associated with disease 7. However, clinical applications 8 of ^31^P-MRS have yet to see widespread acceptance, principally as a result of the method's low intrinsic signal-to-noise ratio (SNR) (^31^P-MRS has ∼10^−5^x lower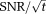 than ^1^H MRI). This has meant that scan times of ∼30 min have been necessary to obtain the important metabolic insights listed above. Even a relatively modest increase in ^31^P-MRS SNR could bring scan times below 10 min and make the method significantly more suitable for clinical studies. Increases in SNR may also permit single-subject comparisons where grouped analysis has previously been required.

Theory predicts that the quality of the raw ^31^P-MRS signal (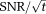) increases approximately in proportion to the scanner's magnetic field strength B_0_ 9,10. Increasing B_0_ thus offers the potential for significant increases in ^31^P-MRS performance, so long as the anticipated gains in ^31^P Boltzmann equilibrium magnetization and radiofrequency (RF) receive sensitivity outweigh any exacerbation of the effects of B_0_-inhomogeneity, RF-induced heating, increased RF thermal noise at the higher field strength and the increased RF power needed to excite the same chemical shift range by the same flip angle 11. At 3T, ^31^P-MRS typically takes ∼30 min to acquire metabolite concentrations in the interventricular septum 12. The spectral SNR at 3T is 2.1× greater than was the case using equivalent methods at 1.5T 13, which has driven a move from 1.5T to 3T for cardiac ^31^P-MRS in recent years. Yet, even at 3T, wider application of ^31^P-MRS has remained seriously constrained by its limited SNR.

Whole-body 7T MRI scanners with ^31^P capability have recently become available commercially, leading several groups to pioneer methods for cardiac MR at 7T (see Moser et al for a review) 14. Theory predicts that cardiac ^31^P-MRS will show a further dramatic (approximately 2.3×) increase in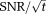 compared with the performance available today at 3T. This gain in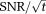 could permit a reduction in scan times allowing the study of dynamic processes; or it could lower variability to allow reliable individual subject comparisons; or it could give sufficient spatial resolution to study focal disease; or it could make visible metabolites that cannot be detected at lower fields, such as inorganic phosphate. With a growing range of techniques having been demonstrated for 7T cardiac magnetic resonance 14, it is now timely to give a proof-of-principle for 7T human cardiac ^31^P-MRS and to test whether 7T ^31^P-MRS truly delivers this predicted data quality in normal volunteers.

We also introduce a novel Look-Locker inversion recovery (IR) chemical shift imaging (CSI) pulse sequence and associated analysis methods to determine the longitudinal relaxation times (T_1_s) of ^31^P-containing metabolites for the first time in the heart at 7T. These T_1_s are required to correct for the effects of partial saturation during postprocessing of data in the main comparison study. We compare the ^31^P T_1_s at 7T with literature values at lower field strengths; and we discuss the origins of the changes we observe.

Thus, we aim to demonstrate a proof-of-principle of 7T human cardiac ^31^P-MRS, to compare the performance at 7T against an established protocol at 3T, and to characterize the longitudinal relaxation times of ^31^P-containing metabolites in the heart at 7T.

## METHODS

### Materials

Scans at 3T used a Trio MRI scanner (Siemens, Germany) equipped with a 10-cm ^31^P Tx/Rx loop coil (PulseTeq, UK). Scans at 7T used a Magnetom 7T scanner (Siemens, Germany), with a T/R switch and preamplifier module (Virtumed, MN), and a purpose-built 10-cm ^31^P Tx/Rx loop coil whose housing has the same subject-coil distance as the 3T coil. Localizer images were acquired at 7T with a separate 10-cm ^1^H Tx/Rx loop coil (Rapid Biomedical, Germany).

The B_1_^+^ profiles and receive sensitivities of the two ^31^P coils were measured using a purpose-built cuboidal phantom comprising 18 L of saline surrounding a height-adjustable 2 × 2 × 2-cm^3^ cube of KH_2_PO_4(aq)_ 15. At a depth of 10 cm [approximately that of the heart 16] and at maximum voltage in vivo, B_1_^+^ was 15.5 μT for 222 V at 3T compared with a B_1_^+^ of 11.1 μT for 270 V at 7T (see Supplementary Information Figs. SI1–3 for details, which are available online). Both coils were fitted with a ^31^P fiducial reference, comprising an 18-mm outer diameter plastic sphere (The Precision Plastic Ball Company Ltd, UK) filled with a solution of phenylphosphonic acid (PPA) and chromium (III) acetylacetonate [Cr(acac)_3_] dissolved in ethanol and sealed with epoxy resin. The concentration of Cr(acac)_3_ was adjusted until initial calibration inversion recovery experiments gave T_1_ ≈500 ms. Both coils also had position markers: 4× cod liver oil capsules at 3T and, at 7T, 2× further plastic spheres of PPA but dissolved in acetone instead to alter the ^31^P chemical shift 17 so as not to interfere with the fiducial reference signal. To confirm that the receive SNR performance of the two coils was comparable, sets of 90° free induction decays (FIDs) with T_R_ ≫ T_1_ were acquired from the cube phantom with the KH_2_PO_4_ source at the same depth (shown in Figure SI4).

Before scanning human subjects, RF heating due to the ^31^P loop coil was calculated following the procedure recommended by the manufacturer (i.e., the procedure in section 4.2.1.2.2 of the document entitled “SAR Parameter N4 B 03” supplied by Siemens.): the quasi-static approximation for a semi-infinite volume of uniformly conductive material was used to determine the ratio of input power to maximum local specific absorption rate (SAR) over a 10-cm^3^ region. This k-value of 5 kg^−1^ (i.e., input in W → SAR in W kg^−1^) was also checked against manufacturer-supplied data for coils of a similar geometry. Finally, following an established protocol 18, these SAR calculations were validated on a meat phantom using fiber-optic temperature probes (Neoptix, UK) to ensure compliance with IEC guidelines during human scans 19.

### Basic Protocol

**Fig 1:**
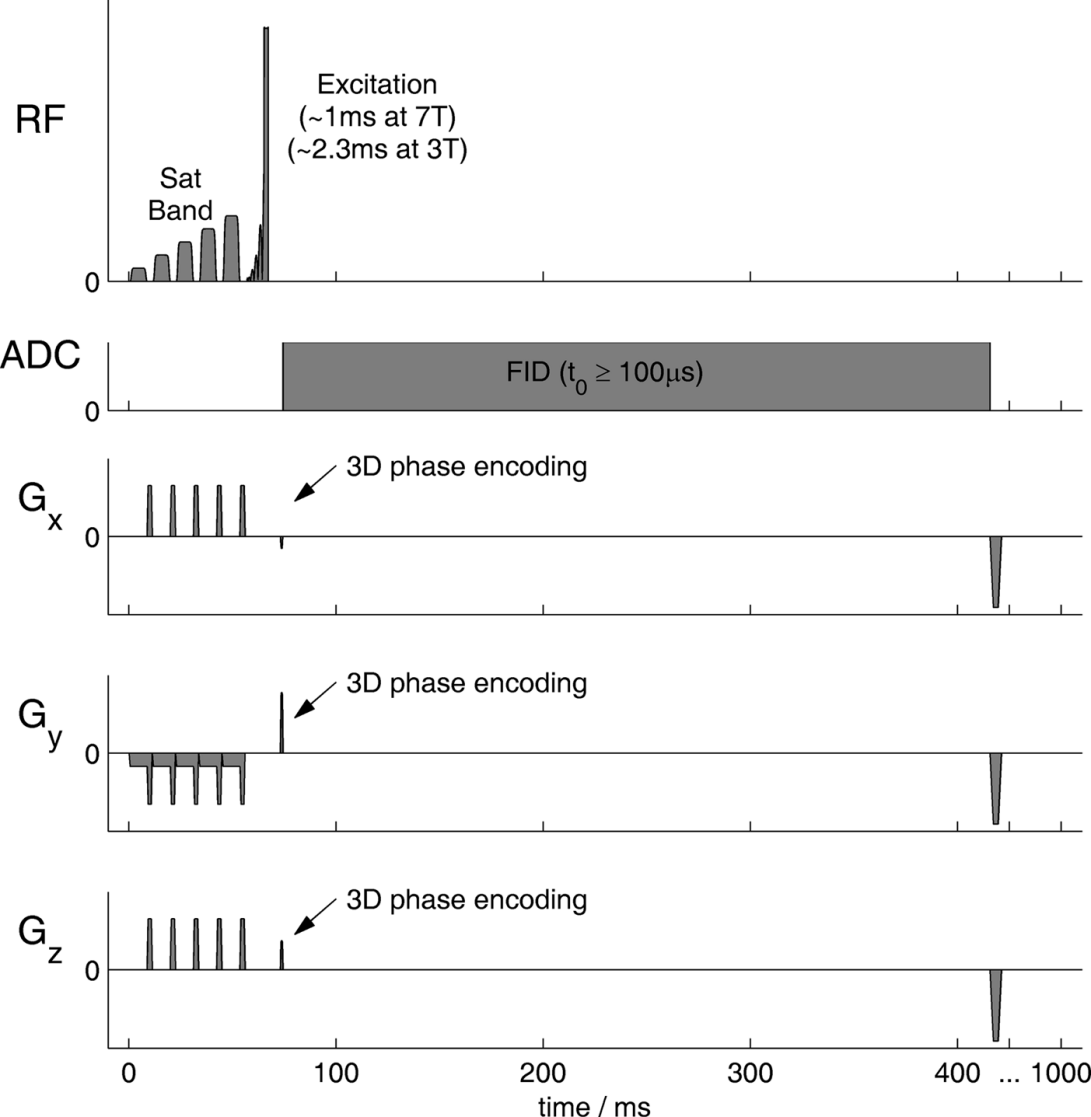
Schematic plot of the UTE-CSI pulse sequence used to record ^31^P spectra. The plot shows a single T_R_, which is repeated typically 1690 times during a scan, setting the phase encoding gradients each time so as to perform acquisition weighted localization. The time t_0_ between the end of each RF pulse and the start of the corresponding ADC block was 100 μs or the duration of the required phase encoding gradient when that was longer.

Normal volunteers were recruited and scanned in compliance with ethical and legal requirements. Spectroscopy acquisitions used the ultra-short-TE chemical shift imaging (UTE-CSI) pulse sequence 13,20 shown in Figure 1. The protocol and sequence parameters were adapted to accommodate the more restrictive limits on peak B_1_^+^ and SAR at 7T, starting from what has been used for several years at 3T 13.

To facilitate the exchange of ^1^H and ^31^P coils at 7T, subjects were scanned supine at both field strengths. Spectroscopy sequences used no gating to avoid the possibility of bias due to increased mis-triggering at 7T 21. “Tune up” shim settings were used for ^31^P spectroscopy at both field strengths.

Localization at 3T was performed with bSSFP and CINE images acquired using the body coil. However, 7T scanners do not have built-in body coils, so localization at 7T was performed using a separate 10-cm ^1^H loop surface coil to record CINE FLASH images with pulse oximeter gating (Siemens, Germany). The ^1^H coil was then removed and replaced with the 10-cm ^31^P loop coil in the same position above the interventricular septum.

At the start of each ^31^P scan, the ^31^P coil was adjusted (match at 3T, tune and match at 7T) for each subject using an RF Sweeper (Morris Instruments Inc., Ottowa, Ontario, Canada) with the coil in situ on the subject. Then, a set of nonlocalized inversion recovery FIDs and a set of images (^1^H bSSFP over 21 slices at 3T, ^31^P FLASH projection images covering three orthogonal planes at 7T) were recorded. From these, the coil location, transmit efficiency (i.e., reference voltage at the primary fiducial) and the coil's B_1_ spatial profile were computed with custom Matlab (MathWorks, Natick, MA) code. Sample output from this code in phantoms is shown on the left of Figures SI2 and SI3.

### T_1_ Determination

The primary focus of this work is to compare the performance of ^31^P-MRS at 7T against 3T. Yet before we can analyze 7T spectra, we need first to determine T_1_s of ^31^P-containing metabolites in the heart at 7T.

Matlab calculations using calibrated B_1_^+^ values demonstrated that the conventional dual-angle method (DAM) 22 would not be appropriate at 7T because B_1_^+^ is insufficient to generate acceptably short BIR-4 or BIRP pulses, and because B_1_^+^ is too inhomogeneous to use hard pulses. Therefore, we created the following novel spectroscopy Look-Locker inversion recovery pulse sequence with adiabatic inversion pulses and chemical shift imaging (CSI) localization.

**Fig 2:**
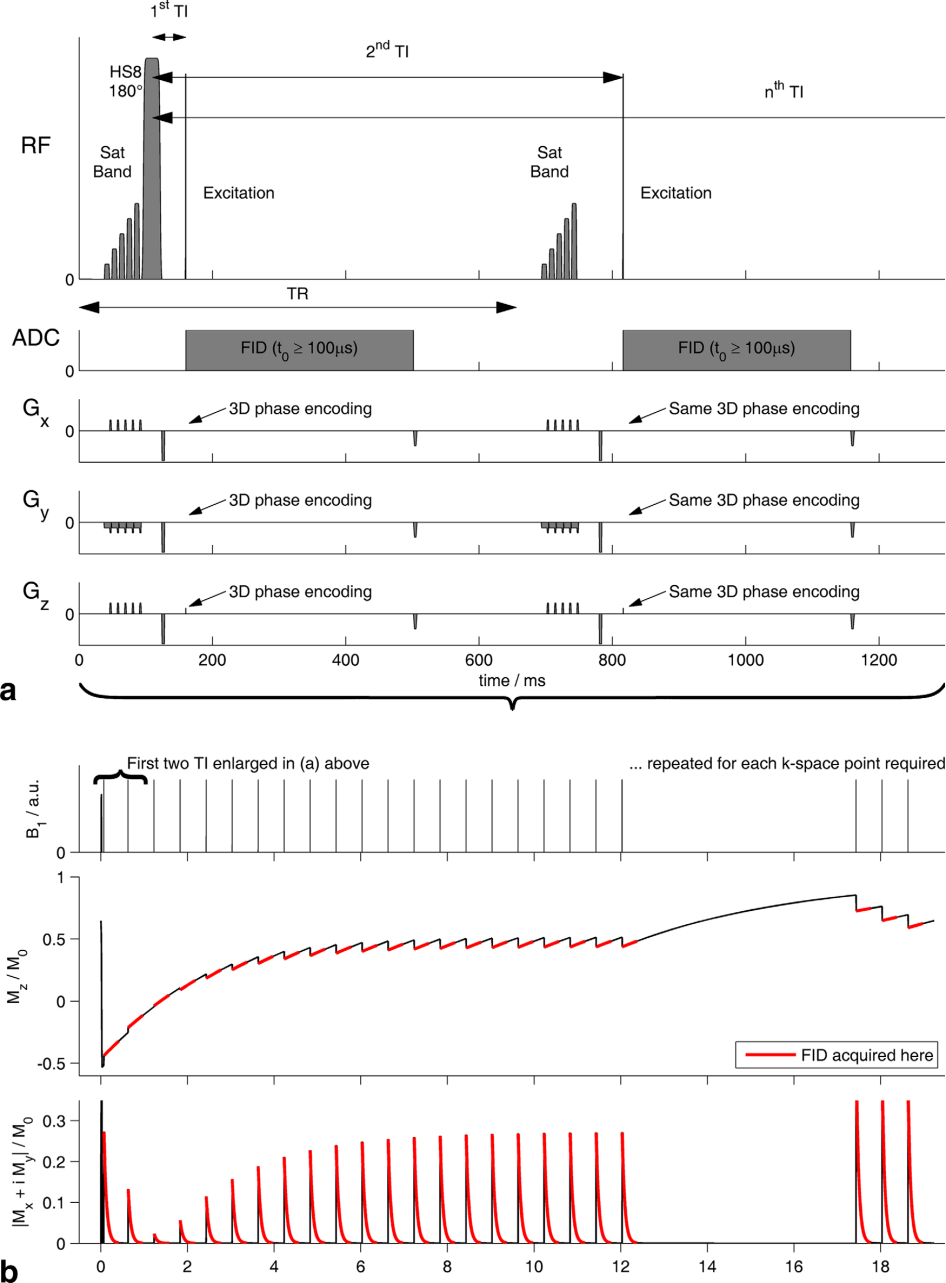
Chemical shift imaging Look-Locker inversion recovery pulse sequence (LL-CSI) used here to measure ^31^P T_1_ relaxation times in vivo. a: Pulse sequence timing diagram showing the first two TI intervals in one T_R_. b: Plots of simulated magnetization evolution during a single T_R_ interval. Each acquisition repeats the RF pulse train in (b) with different phase encoding gradients on each pass according to an acquisition weighted 3D phase encoding scheme. Note how the UTE CSI sequence shown in Figure 1 is used here as a “readout module”. [Color figure can be viewed in the online issue, which is available at wileyonlinelibrary.com.]

Keeping the excitation pulse and three-dimensional (3D) acquisition-weighted k-space sampling strategy from the UTE-CSI sequence (Fig. 1), we extended each T_R_ to include a 4th dimension: inversion time TI. To prepare the magnetization, we added an adiabatic inversion pulse, with parameters optimized in Matlab, and a spoiler gradient. For readout, we acquired data with the same phase encoding and flip angle at TI = 50 ms and at 20× 656 ms intervals thereafter following a Look-Locker scheme 23. We then left a 5.2-s gap to allow magnetization recovery 24, followed by three additional excitations. Pulse voltages were set to run at the SAR limit, prioritizing effective inversion subject to adequate readout SNR. This “LL-CSI” pulse sequence is summarized in Figure 2. The acquisition-weighted k-space sampling pattern is illustrated in Figure SI6.

The LL-CSI sequence was validated by Monte Carlo simulation (see Figure SI8), in phantoms (see Figure SI9) and in human calf muscle where the T_1_s at 7T are known 25,26. Validation data were acquired from the calf muscle of two subjects (male, 21 and 31 years, 70 and 85 kg, 21.6 and 22.8 body mass index [BMI]) over a transverse 6 × 6 × 4 matrix (4 in the HF direction) and a 180 × 180 × 200-mm^3^ FOV, using acquisition weighting with three averages at k = 0. Data were interpolated to an 8 × 8 × 8 matrix by zero-filling in k-space during reconstruction. The pulse voltages were 250 V for inversion and 50 V (15° nominal FA) for readout. The inversion pulse was a 29.7 ms HS8 27 with time-bandwidth product R = 18. To cover all metabolites in a total of 34 min, we performed five repetitions with inversion centered at 836, 47, −566, −1339, and −1589 Hz relative to the readout central frequency, i.e., 586 Hz from PCr. (In units of chemical shift relative to PCr = 0 ppm, these frequencies are equal to +2.1, −4.5, −9.6, −16.0, and −18.1 ppm.)

In the heart, data were acquired from 6 subjects (male, 22–43 years, 68–87 kg, BMI 20.3–26.9) over a transverse 8 × 8 × 6 matrix (6 in the HF direction) over a 240 × 240 × 200-mm^3^ FOV, using acquisition weighting with four averages at k = 0, interpolated to an 8 × 8 × 8 matrix by zero-filling in k-space. The inversion pulse was a 29.7 ms HS8 with R = 24. This long pulse was used to permit inversion of each metabolite despite the considerably weaker B_1_^+^ at the heart. The pulse voltages were 160 V for inversion and 200 V (15° nominal FA) for readout. A saturation band was placed in the coronal plane to suppress signal from skeletal muscle. The saturation voltage was maximized subject to SAR limits, typically at 40 V. In a total of 81 min for each subject, we performed two repetitions with inversion centered at 774 Hz and −827 Hz relative to the readout central frequency, i.e., 586 Hz from PCr. (In units of chemical shift relative to PCr = 0 ppm, these frequencies are equal to +1.6 and −11.7 ppm.)

For each subject, the mid-septal voxel in the most basal short-axes plane showing the papillary muscles was chosen for further analysis. Spectra from that voxel at every TI and from every acquisition on that subject were fitted simultaneously, using the Matlab “lsqcurvefit” routine, to a model function comprising the Fourier transform of a Bloch simulation of the system. The model includes exact RF pulse waveforms and exact timings for RF and ADC events. To remain tractable, the model assumes: that there is perfect spoiling after the inversion pulse and after each readout; that the signal arises from a single point; that J-couplings may be treated with separate peaks that are constrained to have the appropriate relative amplitude and frequency differences for each multiplet component; and that B_0_-inhomogeneity for all the peaks is equivalent to a single exponential damping term exp(-t/ T_2_*). In summary, the model has the following adjustable parameters: T_1_, T_2_, M_0_ and Δν for each of PCr, γ-ATP, α-ATP, β-ATP, and either Pi (leg) or 2,3-DPG (2,3-diphosphoglycerate) (heart) (except PCr where Δν is fixed at 0 Hz); a global T_2_* exponential damping factor; a global phase; and a global B_1_^+^ (Hz V^−1^) factor. The two peaks from 2,3-DPG have separate variable frequency offsets Δν but are constrained to have identical T_1_, T_2_ and M_0_. Fitting required ∼30 min on a Dell Precision T1500 desktop computer with an Intel Core i7 quad-core CPU and 16 GB RAM. Pseudo-code for this algorithm is given in the Supplementary Information.

### Field-Strength Comparison

The performance of ^31^P-MRS at 3T and 7T was then compared in a paired study on nine normal volunteers (male, 22–53 years, 52–88 kg, BMI 19.6–28.7). For each subject, both scans were performed in mornings and within 10 days, to minimize physiological variation. Spectra were recorded using the UTE-CSI pulse sequence (Fig. 1) with a 16 × 16 × 8 matrix, 15 × 15 × 25-mm^3^ (i.e., 5.6 mL) nominal voxel size, acquisition weighting with 10 averages at k = 0 and T_R_ = 1 s. At 7T, excitation was always at the full power supported by the coil (270 V) giving a flip angle of ∼20° in the inter-ventricular septum. At 3T, flip angles were matched approximately to those at 7T using the subject-specific B_1_^+^ maps (akin to the left of Figures SI2 and SI3). Excitation was centered at 250 Hz (i.e., 5.0 ppm) relative to PCr at 3T to ensure a uniform flip angle from 2,3-DPG (at ∼6 ppm) to β-ATP (at ∼−16 ppm) 10. Excitation pulses at 7T were adapted from 3T by reducing their duration by the ratio of field strengths (6.96T / 2.89T = 2.41×) and centering now at 586 Hz (i.e., 4.9 ppm) relative to PCr. A 25-mm-thick saturation band was placed to suppress signal from skeletal muscle in the anterior chest wall. Given the surface coil's inhomogeneous B_1_^+^, we implemented a BISTRO-style saturation scheme 28 comprising 5× HS8 pulses with pulse duration Tp = 8 ms and time-bandwidth product R = 11 27 whose amplitude ramped linearly up to a final pulse at ∼70 V. The exact voltage was set to the maximum permissible given the SAR limits for each subject. The HS8 excitation pulse R and T_p_ were selected to minimize the chemical shift displacement artifact while still giving effective saturation. Bloch simulations, shown in Figure SI7, give a ∼4 kHz bandwidth for the HS8 excitation pulse. Thus, for a saturation band with 25-mm nominal thickness, this equates to a chemical shift displacement of ± 8 mm for Pi and β-ATP.

After each scan, the following analysis was performed using a purpose-made Matlab program. First, the voxel lying at the centre of the interventricular septum (see Figure 4) was extracted for analysis. The spectrum there was fitted using the AMARES 29 implementation in jMRUI v4 30, together with prior knowledge specifying 11 Lorentzian peaks (α,β,γ-ATP multiplet components, PCr, PDE, and 2× 2,3-DPG) and fixed amplitude ratios and scalar couplings for the multiplets. The fitted amplitudes were then corrected for blood contamination by subtracting 30% of the average of the two 2,3-DPG signals from each of the ATP amplitudes 31. The remaining PCr and ATP signals were corrected for the effects of partial saturation 32 using the flip angle at the centre of the voxel, assuming no motion effects and with the T_1_s shown in Table1. The final PCr/ATP ratio is taken as the ratio of the blood and saturation-corrected values of PCr / γ-ATP, discounting the α-ATP peak because it has contributions from NADPH^+^ and the β-ATP peak because it had a phase artifact in some subjects. Finally, the spectral SNR was determined by applying a matched filter and then measuring the SNR as the peak height/baseline SD 33. The final uncertainty in metabolite concentrations was expressed using Monte Carlo error propagation to calculate the Cramer-Ráo lower bounds (CRLB) 34. Statistical comparisons were made in Matlab using paired comparisons wherever possible 35.

**Table 1:** Comparison of ^31^P T_1_s^a^

	Calf muscle 7T (this work, N = 2)	Calf muscle 7T (from (25), N = 8)		Heart muscle 7T (this work, N = 6)	Heart muscle 3T or 2T (literature in Figure 6)	
T_1_ / s						
Pi	6.65 ± 0.23	No value	–	No value	No value	–
2,3-DPG	No value	No value	–	3.05 ± 0.41	No value	–
PCr	3.96 ± 0.07	4.0 ± 0.2	NS	3.09 ± 0.32	5.3 ± 1.7 (N = 50)^b^	^*^^*^
γ-ATP	4.12 ± 0.15	3.3 ± 0.4	*	1.82 ± 0.09	2.8 ± 1.1 (N = 50)^b^	*
α-ATP	1.70 ± 0.02	1.8 ± 0.1	NS	1.39 ± 0.09	2.0 ± 0.9 (N = 5)^c^	NS
β-ATP	1.42 ± 0.12	1.8 ± 0.1	**	1.02 ± 0.17	2.5 ± 0.6 (N = 5)^c^	**
T_2_ / ms						
Pi	58 ± 7	109 ± 17	**	No value	No value	–
2,3-DPG	No value	No value	–	10 ± 6	No value	–
PCr	138 ± 15	217 ± 14	**	220 ± 195	No value	–
γ-ATP	26.3 ± 0.3	29.0 ± 3.3	NS	105 ± 127	No value	–
α-ATP	22.1 ± 0.8	No value	–	14 ± 4	No value	–
β-ATP	18 ± 1	No value	–	70 ± 140	No value	–
+ T_2_^*^ / ms	31 ± 2	–	–	10 ± 4	–	–

a Summary of the ^31^P T_1_s and T_2_s obtained in this study from the calf and heart at 7T. These are compared to literature values also for the calf at 7T to validate our method, and to heart muscle at 3T to demonstrate the significant difference in cardiac ^31^P T_1_s between the field strengths. Statistically significant differences (two-tailed t-test) are denoted ^*^ for *P* ≤ 0.05 and ^*^^*^ for *P* ≤ 0.01.

b These values were recorded at 3T.

c These values were recorded at 2T, no 3T data are available.

## RESULTS

The ^31^P spectra measured from a small phantom (Fig. SI4) show 2.8× higher SNR at 7T compared with 3T, which is close to the ∼2.4× (i.e., 6.96T / 2.89T) B_0_-induced improvement predicted by theory 36. The calculated coil field maps agree with those measured with the adjustable phantom to within 10% (see Figs. SI1–3). These preliminary data confirm that the hardware performs acceptably for ^31^P-MRS at both 3T and at 7T.

Table1 presents a summary of fitted T_1_, T_2_ and T_2_* values from our Look-Locker CSI method applied in the calf muscle alongside pertinent comparators from the literature. Our T_1_s from calf muscle are consistent with those in the literature, giving us confidence that our cardiac T_1_s will be reliable enough for use in saturation correction of the main 3T versus 7T comparison.

**Fig 3:**
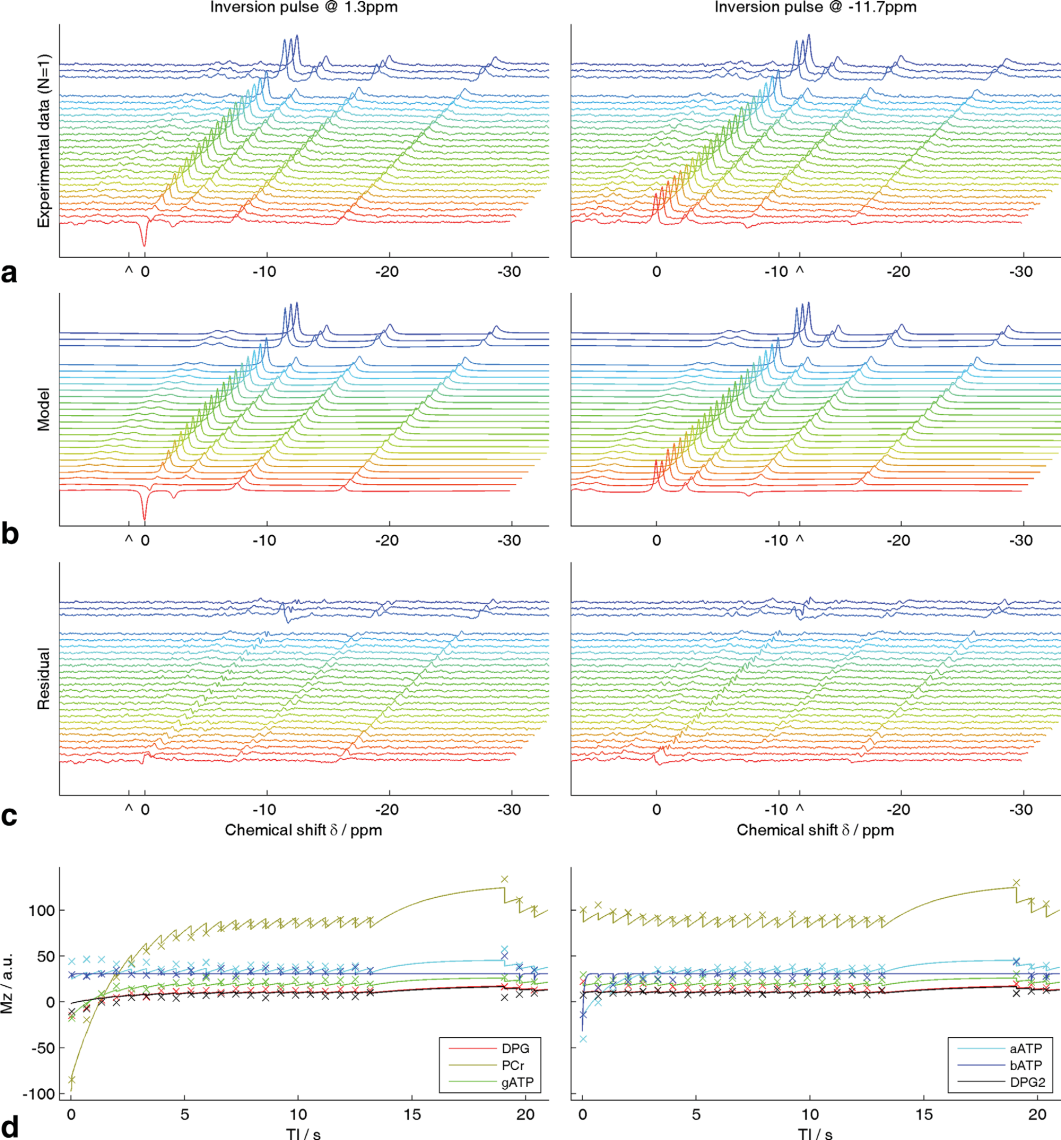
Illustration of ^31^P T_1_ fitting for a typical set of cardiac data. a: Raw spectra from a voxel positioned in the middle of the interventricular septum. Each line shows a different TI, with a break to indicate the equilibrium recovery gap in the pulse sequence. b: Model spectra that were fitted simultaneously to this data with a minimal parameter set as described in the text. c: Residual errors after fitting. Panels a–c are all plotted with identical scaling and “∧” marks the central frequency of the inversion pulse. d: Local maximum intensity extracted around each metabolite peak from the experimental data (Fig. 3a, “x”) and fitted M_z_ (Fig. 3b, lines). These panels are drawn to assist in interpreting panels a–c, but did not form part of the main analysis.

To further illustrate the quality of Look-Locker CSI data in the heart, Figure 3 shows the spectra recorded in one subject to determine cardiac ^31^P T_1_s. Each column corresponds to one execution of the LL-CSI pulse sequence from Figure 2. The inversion and recovery of PCr with inversion centered at 774 Hz (i.e., 1.6 ppm, in the left-hand panel) is evident. The corresponding trace in Figure 3d shows clearly that PCr inversion is reasonably effective. It also shows that there is substantial readout-induced saturation, which confirms the need to include the gap and three additional excitations. The residuals show very little structure, except around the 2,3-DPG peak at short TI, which we attribute to blood flow, and around γ-ATP after the gap, which we attribute to chemical exchange processes (see the Discussion for details).

**Fig 4:**
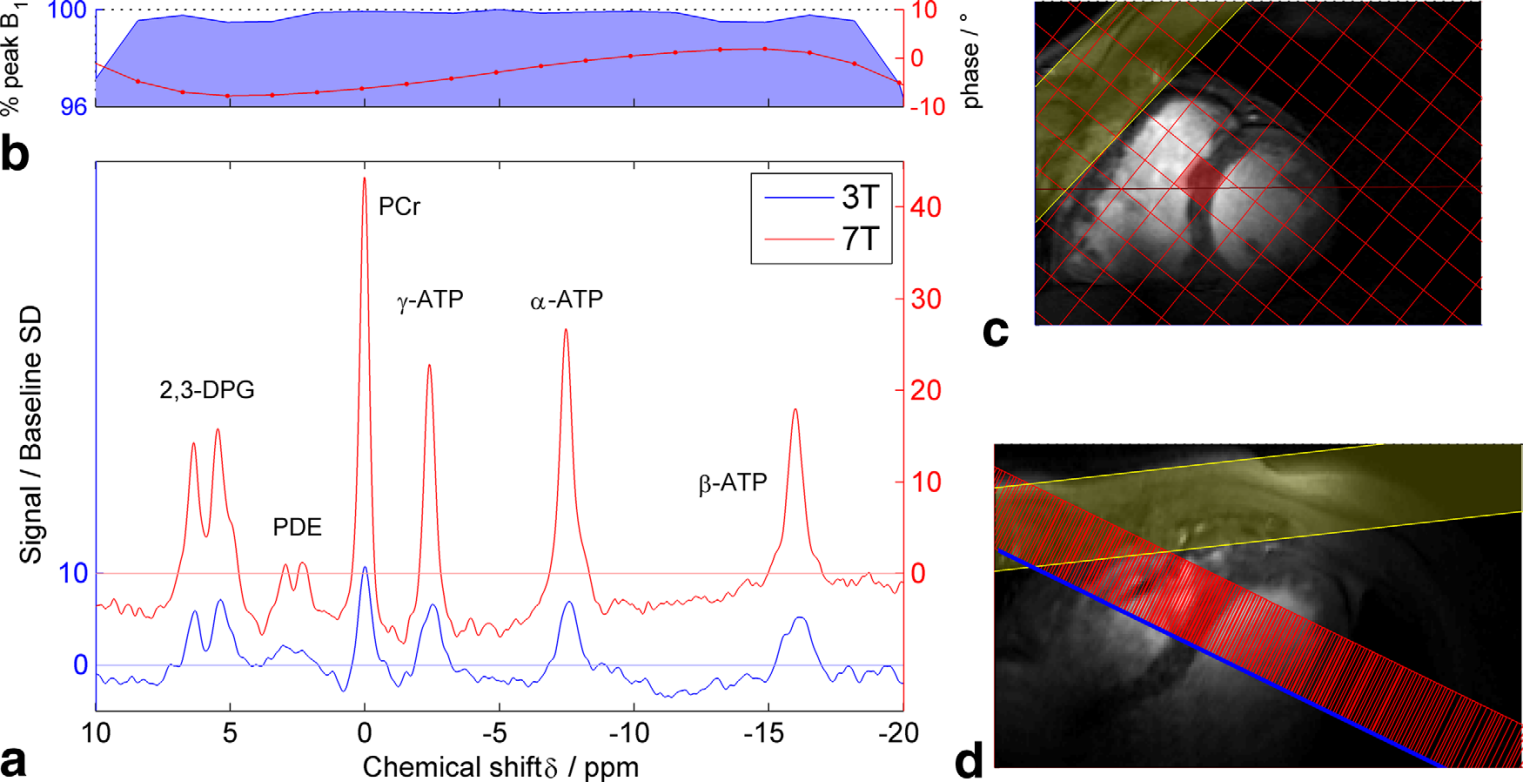
Comparison of spectra in a typical subject at 3T and 7T. a: Real part of the spectra from a voxel in the middle of the interventricular septum. Spectra were apodized with an exponential filter (matched to the fitted PCr linewidth), first order phase corrected and normalized to the resulting baseline noise standard deviation. The peak height of PCr, therefore, shows its SNR according to the standard definition 33. Note that the y-axes are offset for clarity. b: Bloch simulation of the excitation pulse amplitude and phase. This shows that the amplitude is constant to within 1% over the whole spectrum. c,d: The short axis (c) and four-chamber long-axis (d) 7T CINE FLASH localizer images showing the position of the voxel whose spectrum is plotted in panel a. The yellow stripe shows the intersection of the saturation band with the localizer images. The red and blue lines show the intersection of the two localizers. [Color figure can be viewed in the online issue, which is available at wileyonlinelibrary.com.]

Figure 4 illustrates the 3T versus 7T comparison study with the data from one subject. The 7T CINE localizers in Figure 4c,d demonstrate that the quality of localizers attainable at 7T even with a simple 10-cm ^1^H loop coil is quite adequate for cardiac localization. The pulse profile in Figure 4b–note the expanded y-axis scale–shows the uniform excitation generated by the shaped pulse we used. The spectra are shown here after application of a matched filter and normalisation to the baseline SD so that height is proportional to SNR. The increased SNR at 7T is evident. We see also that there is acceptable linewidth and baseline at both field strengths. There is, however, a pronounced signal from blood (2,3-DPG), which Matlab simulations (not shown) lead us to attribute to in-flow effects in the right ventricular blood pool proximal to the coil.

Table2 summarizes the results of AMARES fitting, which completed successfully for all subjects and which detected all the expected metabolites at 3T and at 7T. There is a statistically significant 2.8× increase in PCr SNR and a 2.4× decrease in the Cramer-Ráo lower bounds on PCr concentration. As we would expect in a paired study on normal volunteers, the difference in mean PCr/ATP values (2.1–1.7 = 0.4) is not statistically significant even at the *P* = 0.05 level. Importantly, the PCr/ATP ratio does have a smaller SD at 7T (0.3 at 7T versus 0.5 at 3T) and the CRLB uncertainty in the final calculated PCr/ATP ratio decreases 2.7× at 7T. However, the mean PCr linewidth increases from 16 Hz (i.e., 0.32 ppm) to 37 Hz (i.e., 0.31 ppm) at 7T, which suggests there is scope for further improvement through better optimized shimming.

**Table 2:** Comparison of Cardiac ^31^P Spectra Recorded in Nine Health Volunteers at 3T and 7T^a^

Field strength B_0_	3T (mean ± SD)	7T (mean ± SD)	Ratio 7T/3T	
PCr SNR	11 ± 3	31 ± 13	2.8	**
PCr amplitude CV/%	2.8 ± 1.2	0.6 ± 0.4	0.2	**
Linewidth/Hz	16 ± 2	37 ± 11	2.3	**
Linewidth/ppm	0.32 ± 0.05	0.31 ± 0.09	1.0	NS
Flip angle/ °	20 ± 9	20 ± 5	1.0	NS
PCr SNR (90°, TR ≫ T1)^b^	44 ± 15	105 ± 35	2.4	**
Blood corrected PCr/ATP	1.7 ± 0.5	2.1 ± 0.3	1.2	NS
(Mean CRLB on PCr/ATP)	± 0.67	± 0.25	0.4	–

a Mean values were compared with a paired t-test. Significance is denoted ^*^ at *P* = 0.05, ^*^^*^ at *P* = 0.01.

b This is an extrapolation to the SNR that could hypothetically have been achieved by using 90° excitation pulses and a long repetition time (T_R_ >> T_1_). It removes any effects due to changing ^31^P T_1_s from the comparison.

**Fig 5:**
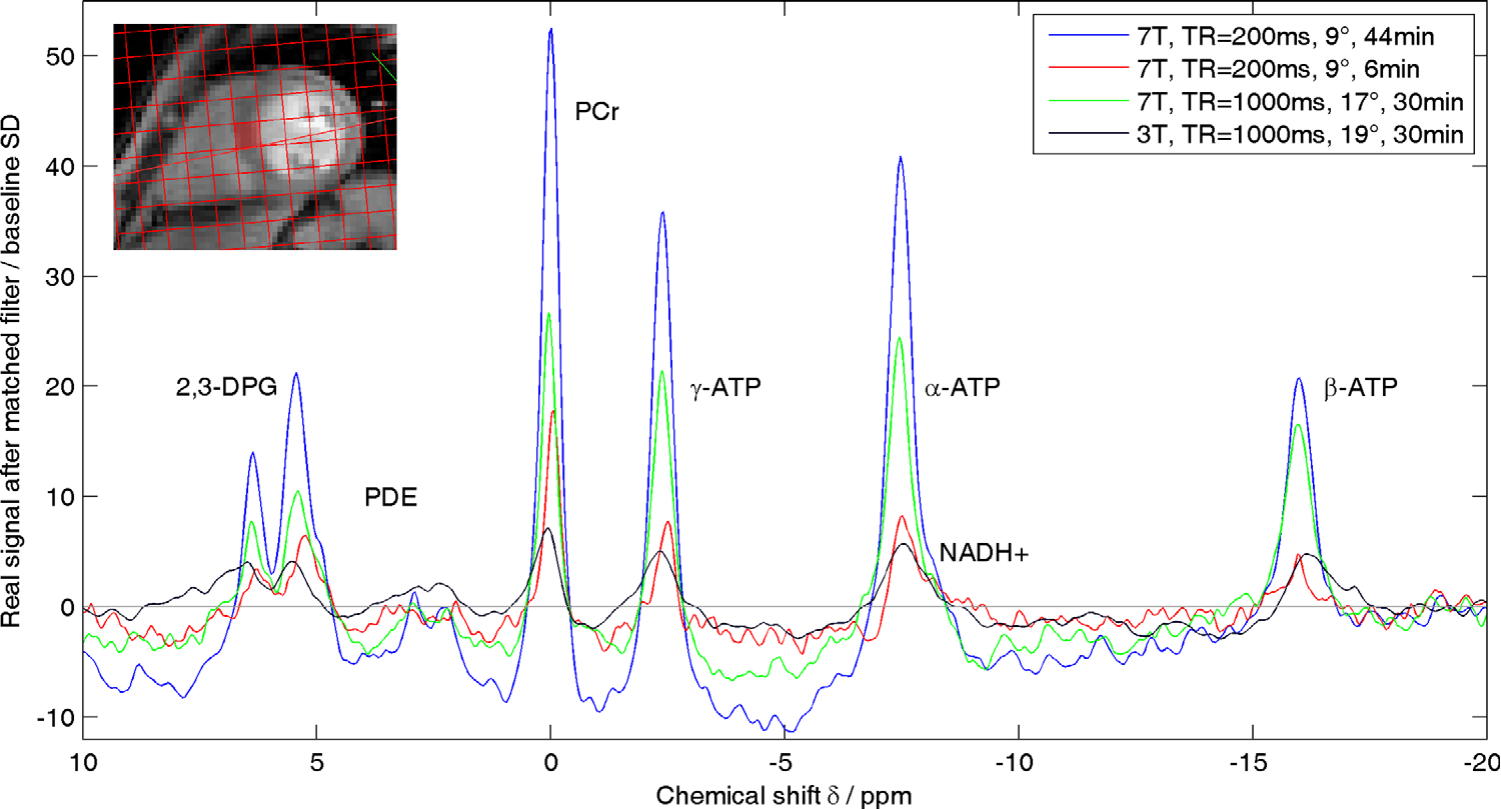
Illustration of the potential gains in SNR or scan duration at 7T compared with 3T. These data were all acquired from the same volunteer from voxels located in the middle of the interventricular septum. The green (7T, T_R_ = 1000 ms, 30 min) line is from the scan used in the main analysis. In comparison, reducing the T_R_ to 200 ms almost doubles the SNR again, or allows a scan with total duration of 6 min (red line) whose SNR is comparable to the 30-min protocol at 3T (black line). [Color figure can be viewed in the online issue, which is available at wileyonlinelibrary.com.]

Finally, Figure 5 compares cardiac ^31^P spectra recorded in the same subject with T_R_ = 200 ms in long and short protocols against the 7T and 3T data described above. This spectrum demonstrates some of the further potential for cardiac ^31^P-MRS at 7T as the methods are refined. The 44-min T_R_ = 200 ms spectrum shows pronounced shoulders to the right of 2,3-DPG and α-ATP which we believe are Pi and NADH^+^. Meanwhile, the 6-min T_R_ = 200 ms protocol gives a spectrum with SNR comparable to that obtained at 3T in our standard 30-min protocol. This shows that cardiac ^31^P-MRS at 7T has great potential to follow dynamic processes such as the response to exercise or pharmacological stressors.

## DISCUSSION

### Validation of LL-CSI T_1_ Method

The SNR in cardiac data that can be acquired in a bearable scan duration (<2 h) is relatively low, so fitting each individual spectrum becomes impractical and it is essential to use our prior knowledge of the metabolite peaks to connect the repeated scans. Our fitting algorithm uses this prior knowledge to give adequate Cramer-Ráo bounds on the fitted T_1_s even with the relatively low SNR data available. Our fitting approach also handles automatically the partial inversion/saturation that is inevitable given the relatively weak B_1_^+^ at 7T.

We validated our acquisition and fitting methods on skeletal muscle, where the T_1_s measured here (see Table1) agree well with those recently reported 25. The exception to this agreement is our longer γ-ATP T_1_ (4.12 ± 0.15 s versus 3.3 ± 0.2 s), which we believe is due to differences in acquisition and a decision in both cases to neglect magnetization transfer in the CK shuttle 37.

We also obtained a value for skeletal muscle γ-ATP T_2_ that agrees well with the literature 25 and report for the first time values for α- and β-ATP T_2_. However, our value for PCr T_2_ is shorter than previously reported 25. Sensitivity analysis of the Bloch simulator shows that both B_1_ and T_2_ govern the efficiency of the inversion pulse while B_1_ also governs the readout-induced saturation that recovers during the “gap” before the final three excitations. Hence, T_2_ is determined by the balance between inversion efficiency and readout-induced saturation; the additional T_2_* factor accounts for any observed linewidth that is not already accounted for by the fitted T_2_. Together, these factors mean that we are sensitive only to T_2_s that are comparable to the duration of the inversion pulse (i.e., 29.7 ms) and also significantly longer than the additional T_2_* factor, which explains our anomalously low value for PCr T_2_ in skeletal muscle and our failure to obtain consistent T_2_s in the heart.

### LL-CSI T_1_ in the Heart

**Fig 6:**
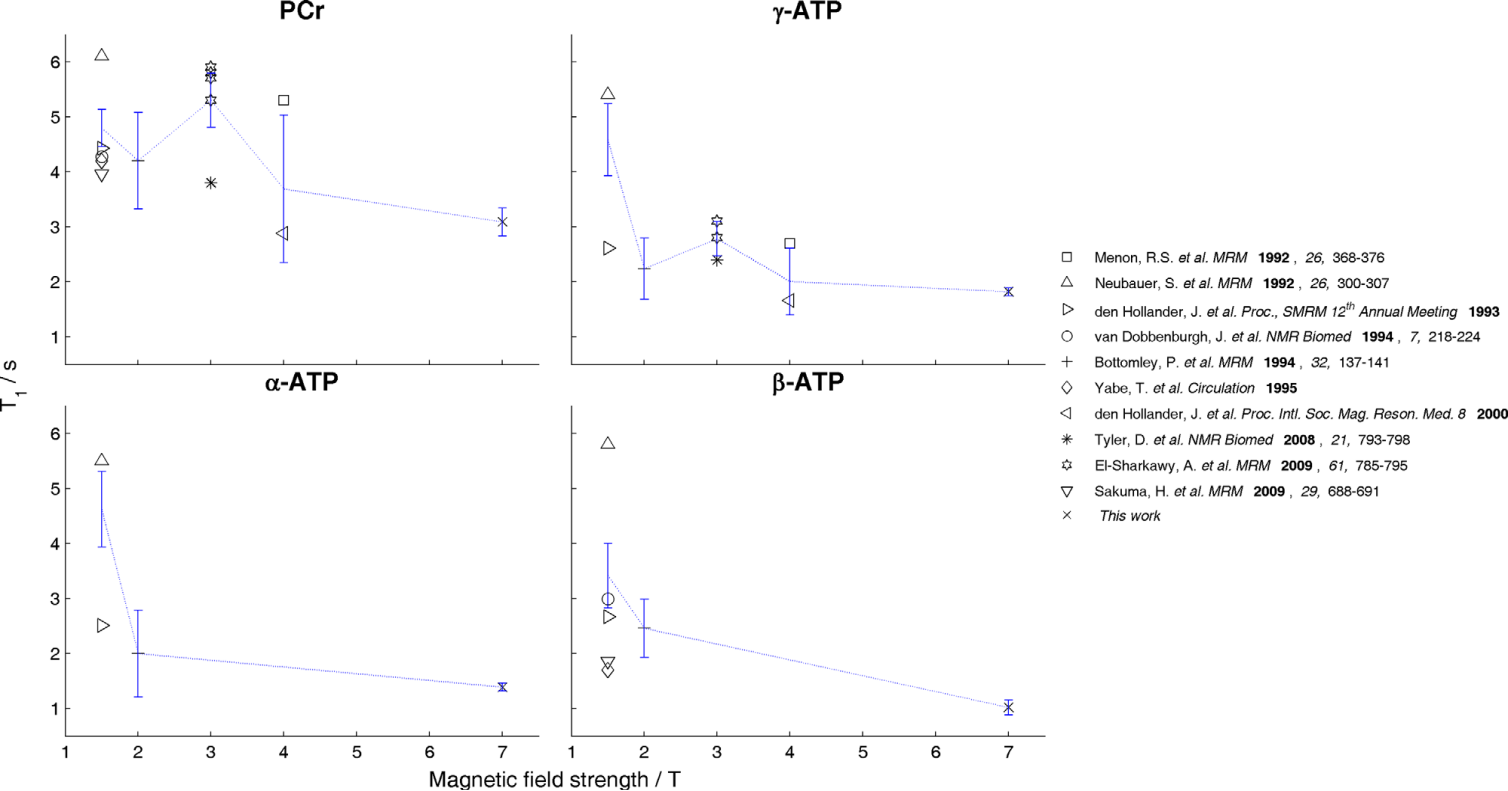
Summary of ^31^P T_1_s reported for PCr and ATP in the human myocardium, grouped by field strength. All relevant data are included from studies published since 1985 that were found with a PubMed, Web of Science or Google Scholar search for the terms “(31 SAME P),” “phosphorus,” “(MRS or spectroscop*),” “cardiac,” and “T1” in various permutations. One mark is shown for each method reported; data from the same source have identical markers. Weighting according to the number of study participants, the average and standard deviation of the combined values are shown for each field strength (to guide the eye, a dashed line connects the means). Full citations for these papers are references 13,18,50,57–63. [Color figure can be viewed in the online issue, which is available at wileyonlinelibrary.com.]

At 7T, T_1_s are shorter in the heart than in skeletal muscle (Table1). Our 7T T_1_s are also significantly shorter than average values reported in the literature for PCr and γ-ATP at 3T and for α,β-ATP at 2T (see Figure 6). This decrease in T_1_ continues a trend tentatively described at lower field strengths 38 and is consistent with animal studies 39.

Proton T_1_s typically increase with field strength B_0_, so the decrease of ^31^P metabolite T_1_s may at first seem surprising. To understand this, we recall that T_1_ relaxation is driven by various mechanisms. Typically dipole-dipole (DD) relaxation dominates (T_1_ ∼ B_0_^3/2^), then chemical shift anisotropy (CSA) (T_1_ ∼ B_0_^−2^), followed by smaller contributions from spin-rotation, scalar coupling, etc. 40,41. The relative contributions from these mechanisms depend on the electronic structure and rotational motion of the molecule in question. The decrease in ^31^P T_1_s with increasing B_0_ suggests a substantial contribution from CSA-driven relaxation. The exact explanation for the T_1_s of PCr and ATP in vivo has been the subject of some debate, with the CSA mechanism being proposed as early as 1985 in rat muscle 39 and investigated in vitro in the early 1990s 42–44 before being invoked to explain data for the T_1_ of human skeletal muscle at 3T 45. The current situation is summarized by Nabuurs et al 46,47.

The analysis above did not consider the significant effects of chemical exchange in the creatine-kinase cycle on T_1_ 48,49. In our cardiac studies, we chose long T_p_ = 29.7 ms, R = 24, HS8 inversion pulses for LL-CSI to maximize inversion efficiency across all metabolites despite the low peak B_1_^+^ at the heart. This meant that we did not invert PCr and γ-ATP selectively, and therefore that there were multiple sets of CK-flux and intrinsic T_1_ values that fitted the data, between which we could not distinguish. Nevertheless, our observed T_1_s are appropriate for saturation correction.

### Limitations of LL-CSI T_1_

Finally, our Look-Locker spectroscopy method is not without its limitations. The current protocol requires an 80-min acquisition. In common with other approaches to determine ^31^P T_1_s, we assume in postprocessing that all signal arises from a region with uniform B_1_, T_1_s, T_2_s, etc., except for an exponential T_2_* relaxation term to account for B_0_-inhomogeneity. The cardiac data sets have insufficient SNR to determine precisely this T_2_* value and the individual metabolite T_2_s, essentially because the data do not sufficiently constrain the fitted linewidths. Also, it was not feasible to consider the in-flow of blood, so the “T_1_” we observed for 2,3-DPG is likely to be an underestimate and there may be small artifacts due to blood contamination of the ATP resonances.

### 3T versus 7T Comparison

This study reports the first human 7T cardiac ^31^P spectra. It also provides the first quantitative matched comparison of the performance of human cardiac ^31^P-MRS at a field strength exceeding previous reports at 3T 12,13 and at 4T 50,51. Figure 4 and Table2 show the marked superiority of cardiac ^31^P spectra at 7T relative to 3T. The SNR of every peak increases and therefore the quality of the ensuing AMARES fits also increases, e.g., the CV of the PCr amplitude decreases by 2.4×.

Table2 shows an extrapolation of the PCr SNR to a hypothetical fully relaxed acquisition with 90° excitation flip angle, revealing a 2.4× gain at 7T. Meanwhile, in this study, PCr SNR increased by 2.8×. Together, these values demonstrate that, with this protocol, there is not only a 2.4× improvement arising from the increase in B_0_ alone as predicted by theory 9,36 but also a further 1.2× improvement because of better M_z_ recovery due to the decrease in PCr T_1_ (see also Table1).

The 10-cm Tx/Rx coil used in this study was chosen to facilitate a fair comparison between field strengths. However, it is unlikely to be optimal design for cardiac ^31^P-MRS either at 3T or at 7T. In particular, a 10-cm loop may not be appropriate for female subjects or those with a high BMI. A loop coil has optimal SNR at a depth of approximately 1× its diameter, making a 10-cm coil most appropriate for lean male subjects. A more sophisticated 7T coil, e.g., using a larger Tx loop combined with one or more <10-cm Rx elements 13,52–56, should give even better data and facilitate the transition of 7T ^31^P-MRS to clinical populations.

In this study, we chose to match the flip angle at 3T to that at 7T because our maximum flip angle at 7T was limited by the maximum rated power of the coil T/R switch. Furthermore, there is no direct link during coil design between achievable B_1_^+^ and RF heating or peak RF power requirements. Hence, it would not have been possible to guarantee an “optimal” coil design at both field strengths, precluding such a comparison. Similarly, we chose not to use gating in this study, primarily to avoid potential difficulties at 7T. As we gain more experience at 7T, the gating “problem” is becoming less acute with better subject preparation and ECG positioning, etc. We know that there is scant difference between ECG gated and nongated 3D CSI ^31^P-MRS data quality at 3T (unpublished data), so we opted to eliminate this potential complication, because it does not inexorably diminish the long-term capabilities at 7T.

The SNR improvement with field strength varies noticeably between subjects (e.g., PCr SNR increased 0.8, 1.1, 2.4, 2.9, 3.5, 3.9, 4.1, 4.2, and 4.3×). We attribute this to the challenge of accurate coil positioning with a small loop coil. When performing the comparison study, we checked the coil position images and accepted a position within 1 cm of the target position on the chest. Even if optimally placed, a 10-cm Tx/Rx loop will show significant variation in receive sensitivity and B_1_^+^ homogeneity at the interventricular septum due to variations in body shape and in the coil, septum distance. The fraction of the voxel filled with myocardium will also vary between subjects. These effects are consistent with previous reports regarding the positioning of ^31^P-MRS surface coils 13,54.

Cardiac ^31^P spectra typically contain a contribution from blood in addition to the desired signal from the myocardium. Blood contamination gives rise to the two 2,3-DPG peaks at ∼6 ppm and to additional ATP signals which overlap myocardial ATP. We therefore subtract 30% 31 of the mean 2,3-DPG peak amplitude from each ATP peak to remove contributions to ATP from blood. It is not immediately apparent whether this blood correction should be performed *before* or *after* saturation correction. The correct choice depends on the degree of replacement of blood by in-flow during each T_R_: there is almost 100% in-flow in the ventricular blood pool, but much less in the myocardial capillary bed. Given the size of our voxels, we believe ventricular blood dominates and, therefore, we perform blood correction before saturation correction. This also avoids the difficulties in obtaining a meaningful T_1_ for 2,3-DPG which were discussed above.

Linewidths previously reported at 3T for PCr are on the order of 22 ± 12 Hz (i.e., 0.44 ± 0.24 ppm) 13, which compares favorably with the data reported in Table2. In this study we only used “tune up” B_0_ shims because of difficulties with ^1^H image-based shimming at 7T due to the inhomogeneous B_1_ from the ^1^H loop. Therefore, our 7T ^31^P linewidths represent an (encouragingly good) worst case for future studies.

Finally, Figure 5 gives a glimpse of the great promise for cardiac ^31^P-MRS at 7T. Being able to obtain spectra with acceptable SNR in 6 min will open the way to more detailed study of the response of ^31^P energetics to stressors such as exercise or infusion with dobutamine. Three 6-min scans could easily be incorporated into a more general protocol. Alternatively, one could follow the time course of recovery in 6-min intervals allowing more detailed characterization of the recovery curve than current paradigms. At the other extreme, the 44-min 200-ms T_R_ spectrum suggests that it may soon be possible to quantify further metabolites in a cardiac ^31^P-MRS scan, extending the options available for clinical research. Finally, the almost 10× improved SNR comparing the 44-min 200-ms T_R_ spectrum against the 30-min 3T spectrum could be used to increase the resolution of the 3D CSI matrix by a factor of exp(ln(10) / 4.5) = 1.67 while still obtaining adequate SNR for further analysis. This would offer greater chances of detecting regional energetic anomalies as opposed to the global changes we are sensitive to today.

## CONCLUSIONS

We have demonstrated proof-of-principle for cardiac ^31^P-MRS at 7T. We also quantified the significant improvements in spectral SNR relative to the previous gold-standard field strength of 3T (e.g., PCr SNR increased 2.8×). We have also developed a new Look-Locker CSI spectroscopy method and used it to determine for the first time the T_1_s of high energy phosphate metabolites in the human heart at 7T. These cardiac 7T T_1_s were found to be lower than values reported in the literature at 3T and lower than in skeletal muscle (e.g., PCr T_1_ is 3.09 ± 0.32 s at 7T in the heart versus 3.96 ± 0.07 s in skeletal muscle at 7T, and versus 5.3 ± 1.7 s at 3T in the heart). Together, the increases in Boltzmann magnetization and spectral bandwidth at 7T coupled with the decreases in T_1_, provide a substantial boost to the sensitivity of cardiac ^31^P-MRS at ultra-high field strengths. We were able to acquire spectra in 6 min of a quality that took 30 min at 3T. Alternatively, we would be able to increase the CSI resolution by ∼1.67× while maintaining adequate spectral SNR.

To close, we have found that cardiac ^31^P-MRS shows great potential at 7T. We recommend 7T as the field strength of choice for applications of ^31^P-MRS to clinical research.
