# Supplementary material for: Human cardiac 31P magnetic resonance spectroscopy at 7 tesla
Source: Magn Reson Med. 2013 Sep 4;72(2):304–15. doi: 10.1002/mrm.24922 (PMC4106879; doi:10.1002/mrm.24922)
Supplement: Supplementary file 1 — Supporting Information [file mrm0072-0304-SD1.docx]

Human cardiac ^31^P magnetic
resonance spectroscopy at 7 Tesla:
Supplementary Information

# Coil B_1_^+^ calibration


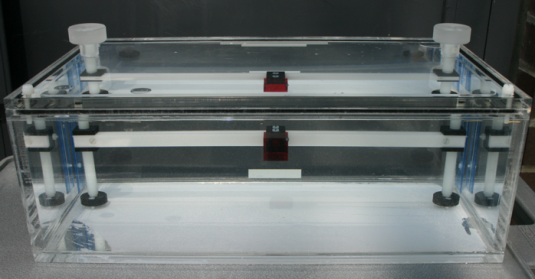


**Figure SI1**: Photograph of the phantom used for B_1_^+^ calibration. The phantom comprises an 18L cuboid filled with physiological saline (73mM NaCl) in which is immersed a 2x2x2cm^3^ cube of KH_2_PO_4_ solution from which the ^31^P signal arises. The coil is centred directly above the cube using the scanner's laser position indicator. Two thumb screws then allow adjustment of the distance between the coil and the KH_2_PO_4_ cube without moving the coil. The KH_2_PO_4_ in the cube has a ^31^P reference T_1_= 2.00s at 3T and T_1_ = 1.81s at 7T.


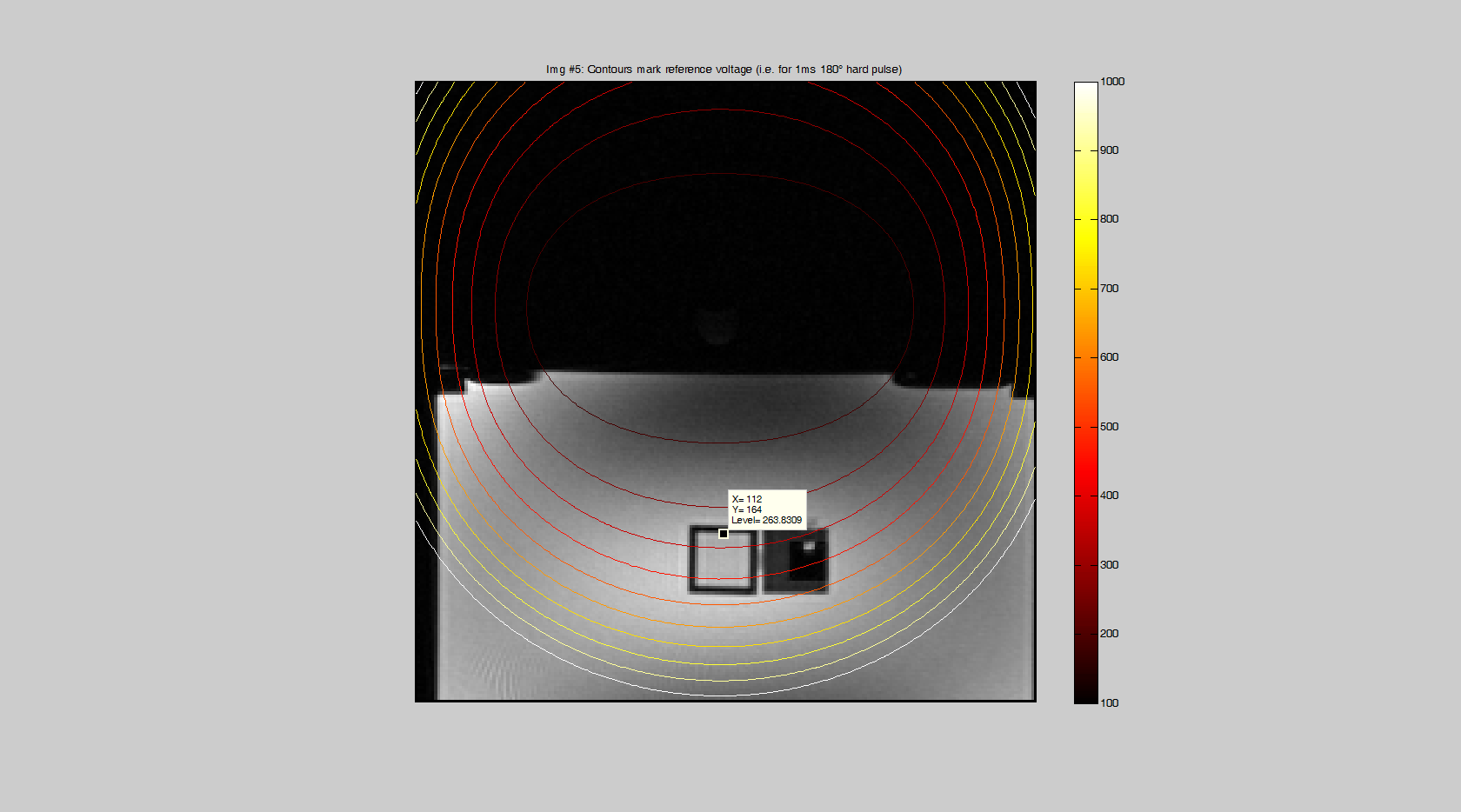

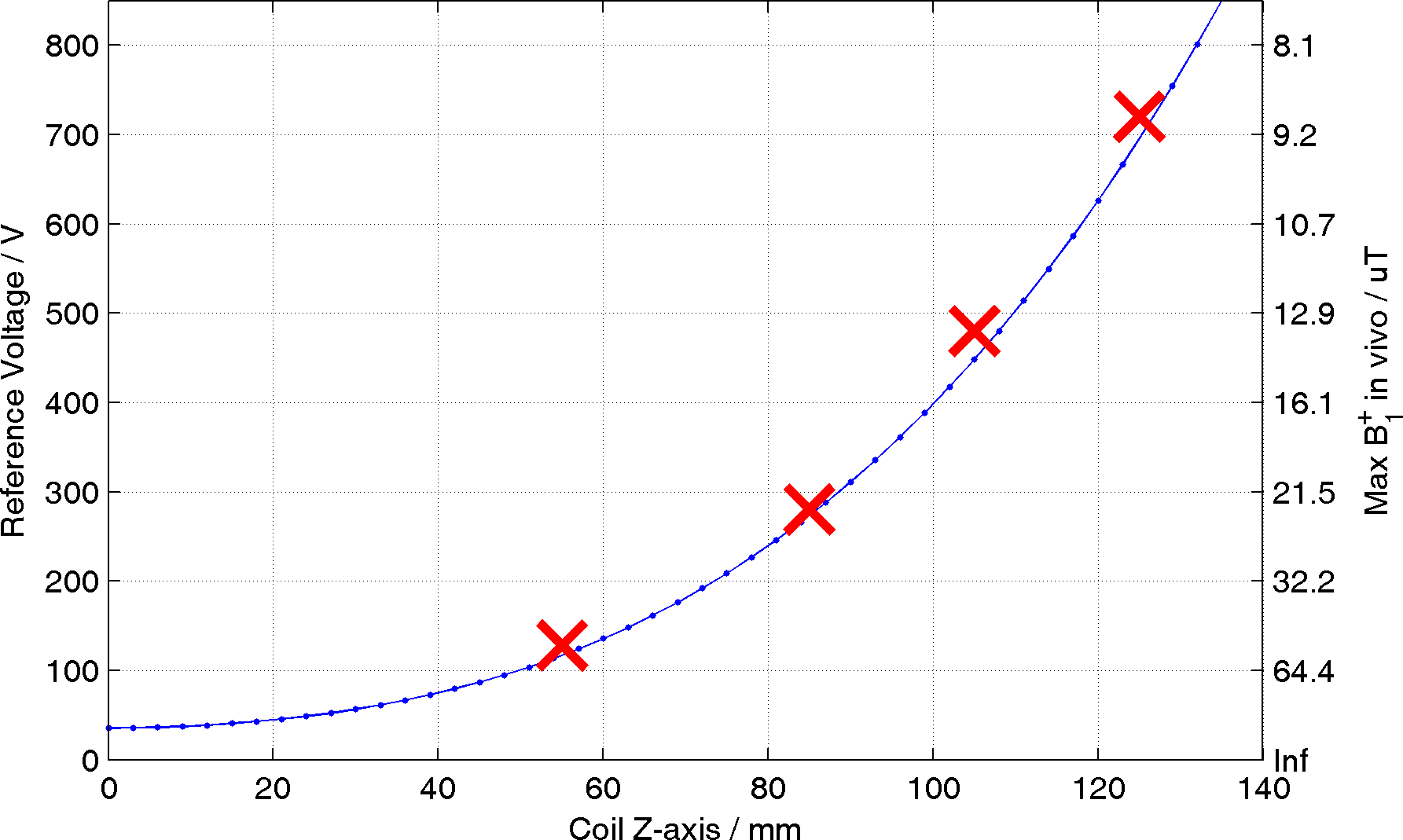


**Figure SI2**: Experimental validation of coil field maps for the ^31^P loop at 3T using the phantom in Figure SI1. Left: "Reference voltages" (i.e. the voltage required for a 1ms hard pulse to yield a nutation of 180°) calculated from IR FIDs at the reference fiducial extrapolated with a Biot-Savart law calculation of B_1_^+^. Right: The blue line shows the calculated reference voltage (from the left figure) along a line through the coil centre. The red x denote direct measurements of B_1_ in the cube by repeated acquisition of FIDs with 4ms excitation, 1500ms T_R_, 4 preparation scans and 4 averages followed by fitting using the phantom T_1_ previously determined in fully relaxed inversion recovery experiments. There are no scaling parameters in this calculation. The Biot-Savart calculated B_1_^+^ values are accurate to within 9%. The "Max B_1_^+^ in vivo" values were limited by the coil's maximum rated voltage.

**
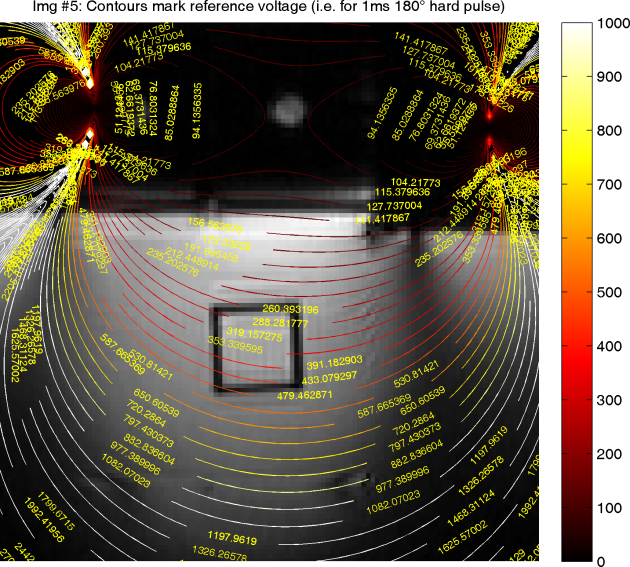

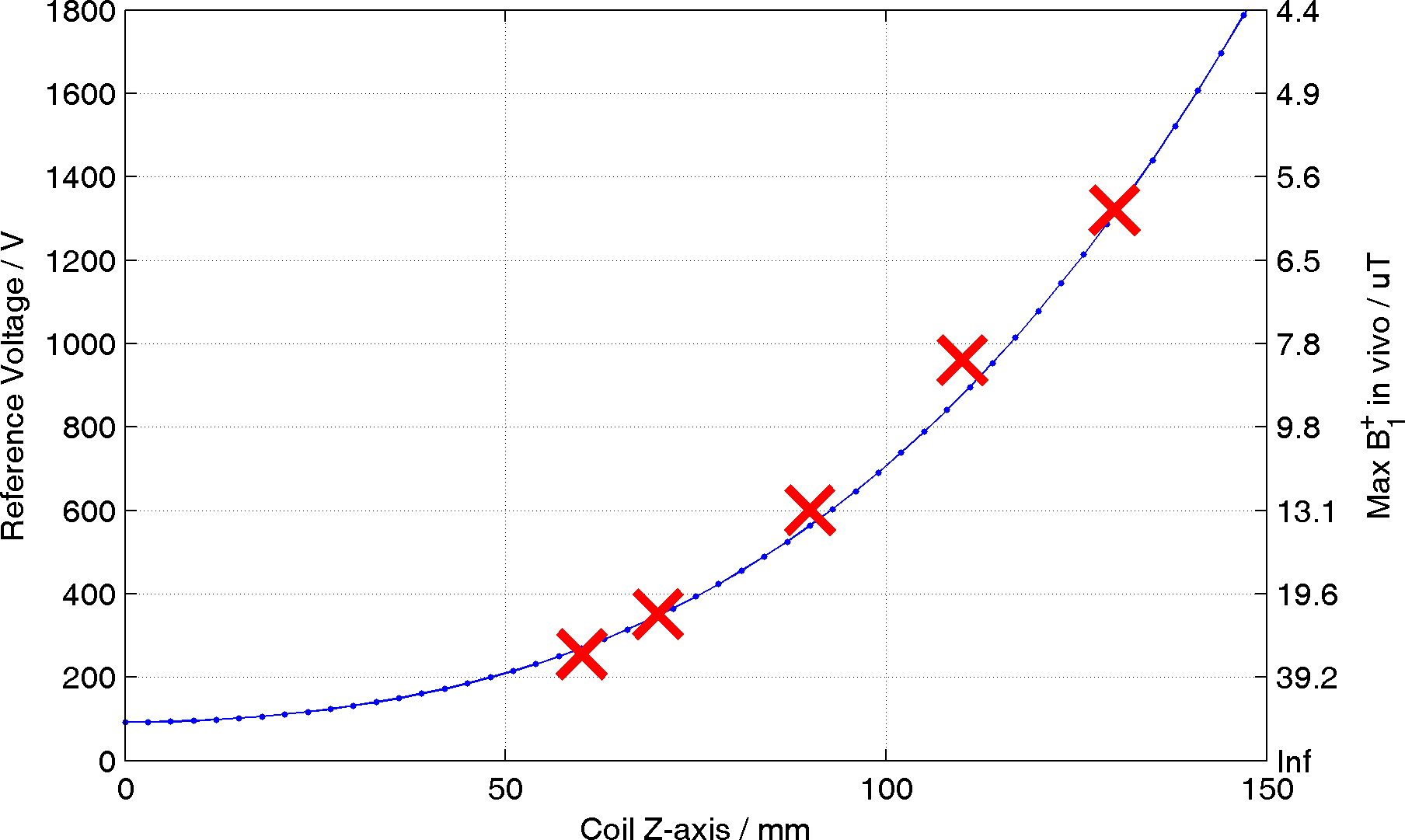
**

**Figure SI3**: Experimental validation of coil field maps for the ^31^P loop at 7T using the phantom in Figure SI1. Left: Calculated reference voltages extrapolated using the Biot-Savart Law from the value found by IR FIDs at the reference fiducial . Right: The blue line shows the calculated reference voltage (from the left figure) along a perpendicular line through the coil centre. The red x show measured 180° null voltages from unlocalized FIDs fitted as in Figure SI2. There are no scaling parameters in this calculation. The Biot-Savart calculated B_1_^+^ values are accurate to within 10%. The "Max B_1_^+^ in vivo" values were limited by the coil's maximum rated voltage.

# Phantom validation of coil SNR


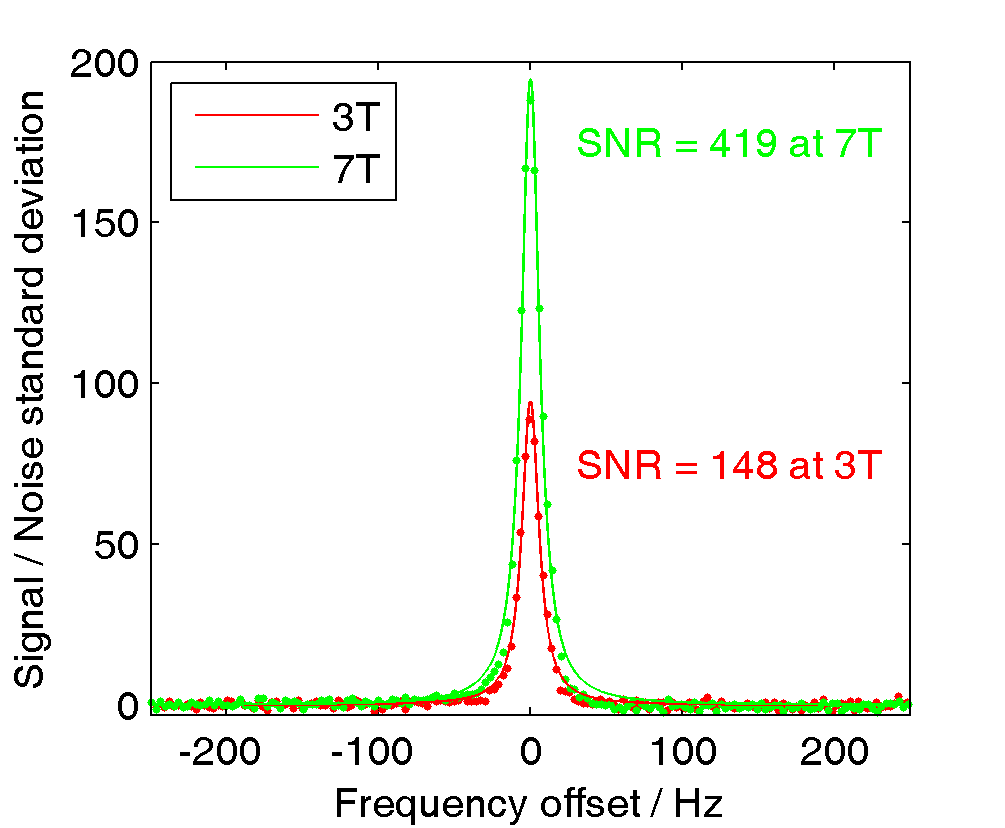


**Figure SI4**: Comparison of the signal-to-noise ratio on the same phantom at 3T and 7T. The phantom in Figure SI1 was set to a depth of "3cm" on the scale (i.e. from the inner surface of the perspex box). FIDs were recorded with a range of excitation voltages to locate the first null (i.e. the voltage for a 180° pulse). FIDs were then acquired at half this voltage (i.e. 90° excitation), with 30s TR (i.e. >> 5xT_1_) and with 1 preparation scan and 4 averages. These averaged FIDs are plotted above. Following standard methods , the SNR is defined as the peak height after application of a matched filter divided by the baseline standard deviation.

# Determination of fiducial positions

Correction for partial saturation, which affects each metabolite differently, requires the local flip angle at each voxel. To determine the coil position at 3T, we normally image cod liver oil capsules attached to the coil housing with a ^1^H bSFFP sequence. However, since there is no body coil in a Siemens 7T scanner and our coil was not tuned for ^1^H, it was impossible to use ^1^H imaging to determine the coil position at 7T.

Instead, 3 markers containing phenylphosphonic acid (one of these was also the flip angle reference and had ethanol as solvent, the others used acetone as solvent) were attached to the coil housing and ^31^P FLASH projection images were acquired in three orthogonal planes during a single breath-hold at the start of each experiment. The 3D position of the markers was then determined automatically in the Matlab post-processing as follows:

1. The markers were located in each of the projection images automatically.

2. Each mark denotes the presence of one *or more* fiducials along a line *L*_i_ normal to the projection plane that passes through the mark.

3. The 3D position of a fiducial should be the point of intersection of these three lines. In practice, to compensate for uncertainties in the marker position, we use the point of minimum sum of least squares distances to these lines. This point is determined by the following algorithm.

We define each line *L*_i_ in terms of a unit vector along the line *u*_i_ and a point *p*_i_ in the plane orthogonal to the line *L*_i_ that also contains the origin. The sum of squared distances *d* from the fiducial point x is

$$d=\sum_{i} \left|\left(I-u_{i}u_{i}^{T}\right)x-p_{i}\right|^{2}$$

The value of d is minimised when x is the least squares solution of

$$Ax=b$$

where the 3x3 matrix $A=\sum_{i} \left(I-u_{i}u_{i}^{T}\right)$ and the 3x1 vector $b=\sum_{i} p_{i}$. In Matlab, x is therefore given simply by "x=A\b" where \ is the Matlab "mldivide" operator.

4. It is not certain which marks correspond to one-another in the projection images. We therefore repeat step 3 for each of the 9 possible combinations. The true geometry has the smallest sum of residual distances *d* between normal lines.

This whole procedure takes a few seconds and requires no manual intervention.

**
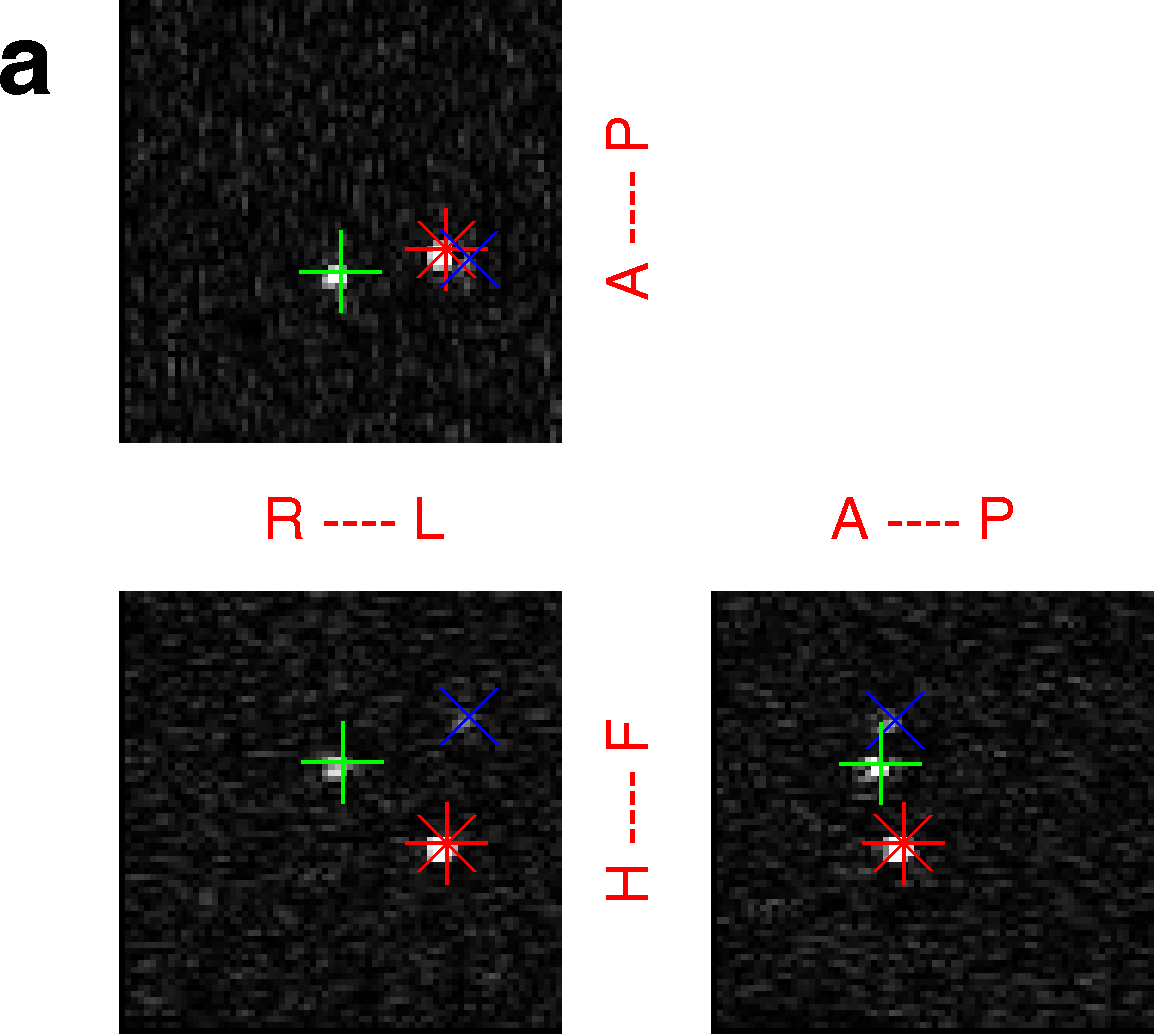

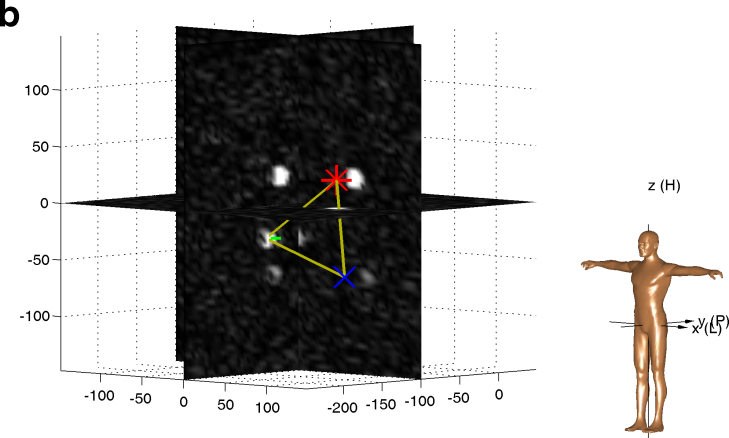
**

**
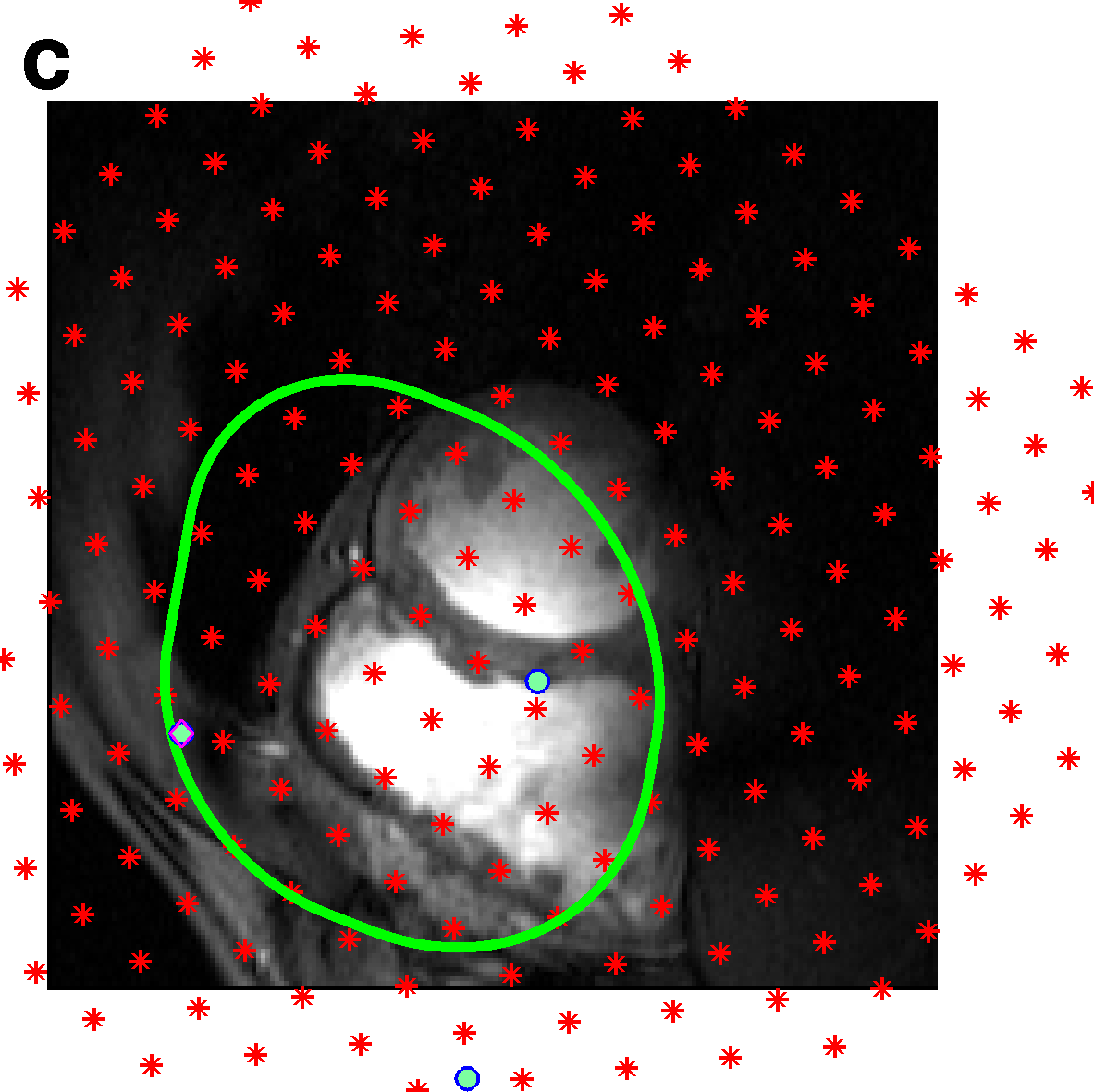
**

**Figure SI5**: Illustration of the results of fiducial position determination at 7T. (a) ^31^P FLASH projection images acquired in 3x7s. (b) Automated determination of the 3D coordinates of the fiducial markers. (c) View of the coil (green line) projected onto a short-axis localizer. Blue points mark the fiducials, red * mark the centres of the spectroscopy voxels. Note that this oblique view of the coil gives the illusion that it has been distorted. However, rotating this 3D view and comparing several orientations confirms that there is in fact no such distortion.


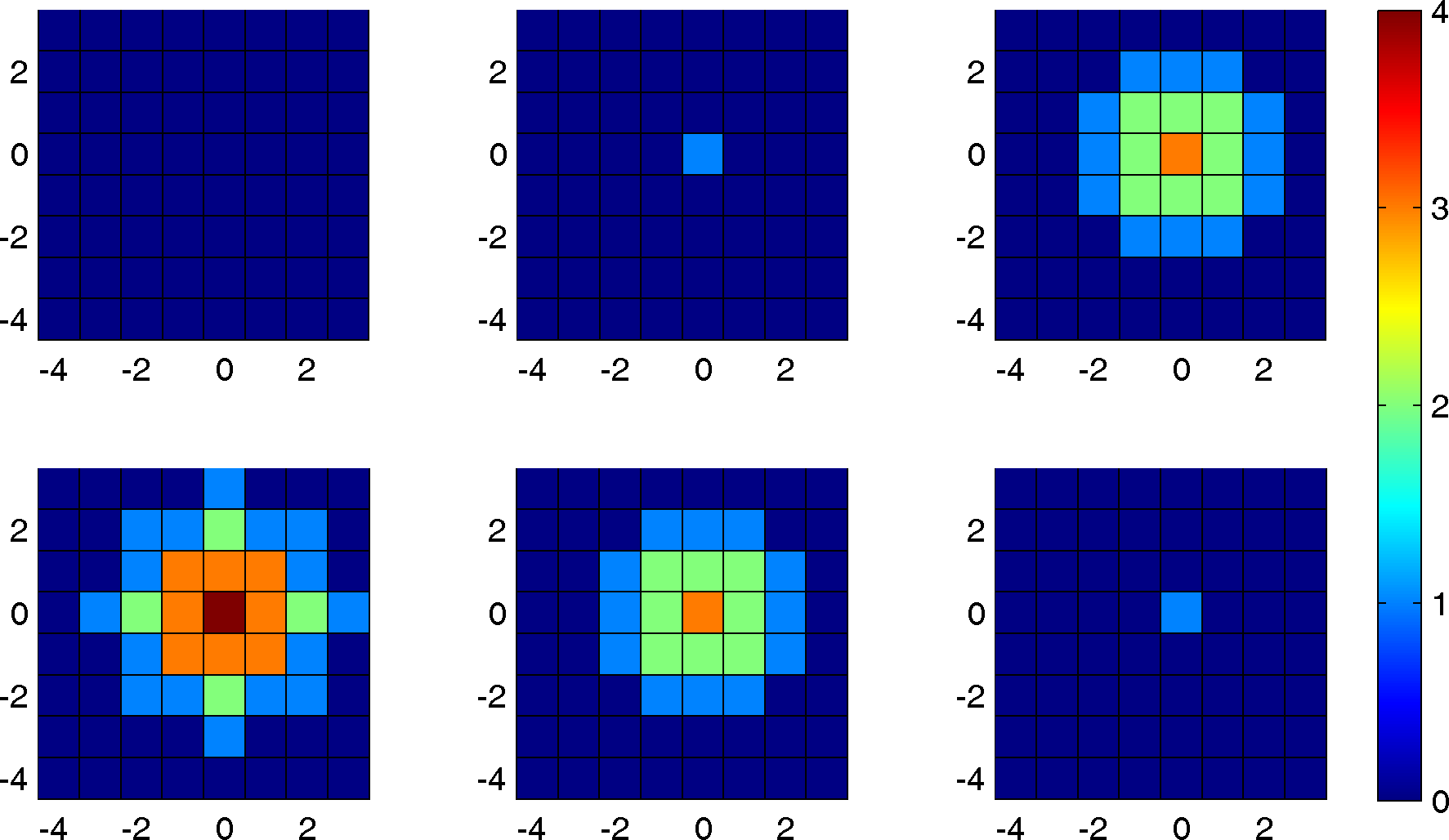


**Figure SI6**: Plot of the k-space sampling pattern produced for an 8x8x6 CSI matrix with 4 averages and acquisition weighting by the Siemens CSI pulse sequence. There are a total of 116 phase encode steps. This compares with 1536 phase encode steps that would be required for full k-space sampling of the same 8x8x6 CSI matrix with 4 averages.


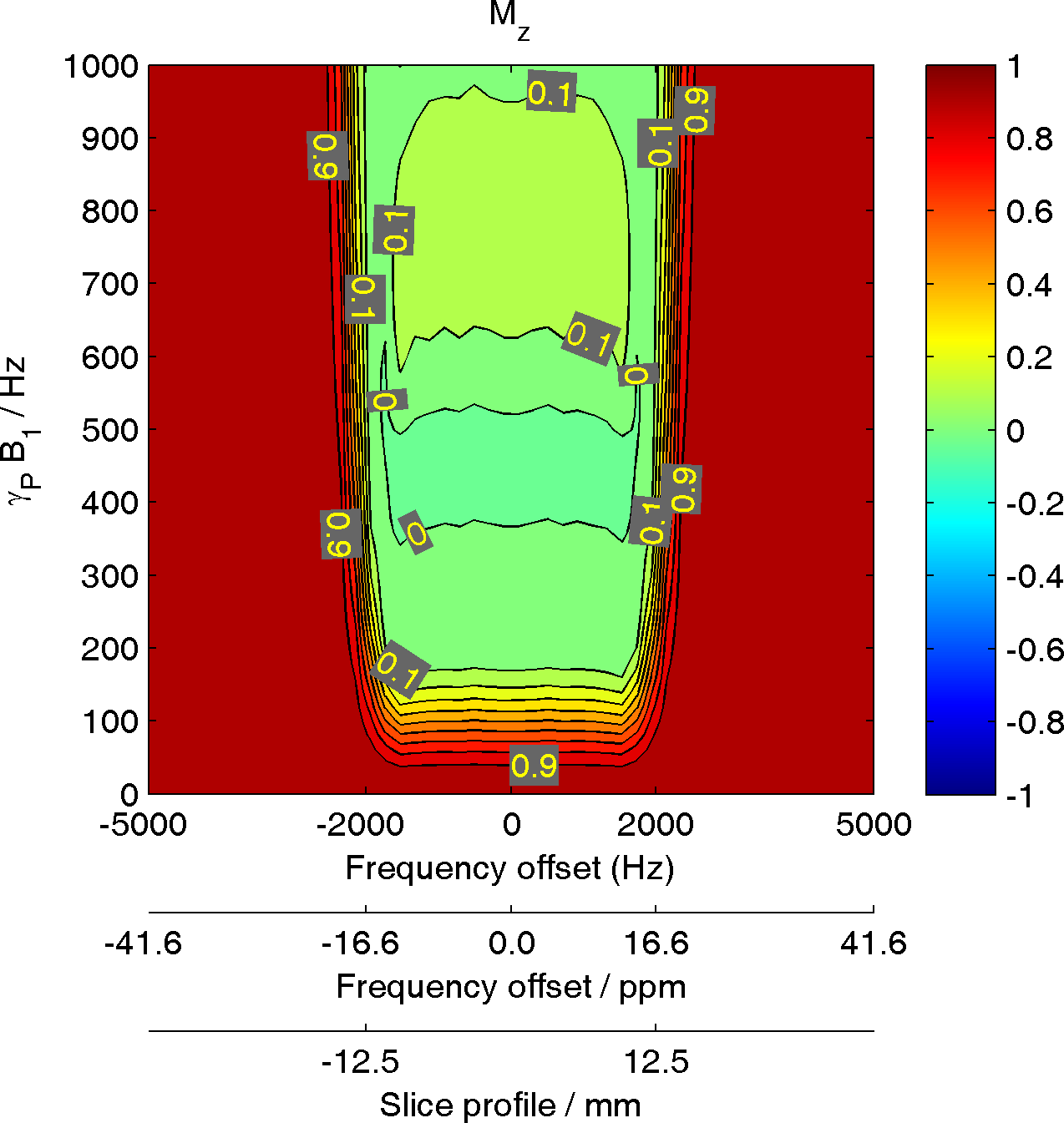


**Figure SI7**: Calculated response to BISTRO-style saturation pulse train performed by numerical solution of the Bloch equations with T_1_=1.835s and T_2_=80ms and assuming perfect spoiling. Three equivalent horizontal axes are marked to show clearly the chemical shift displacement artefacts that are predicted and the slice profile when a 25mm nominal slice thickness has been prescribed.


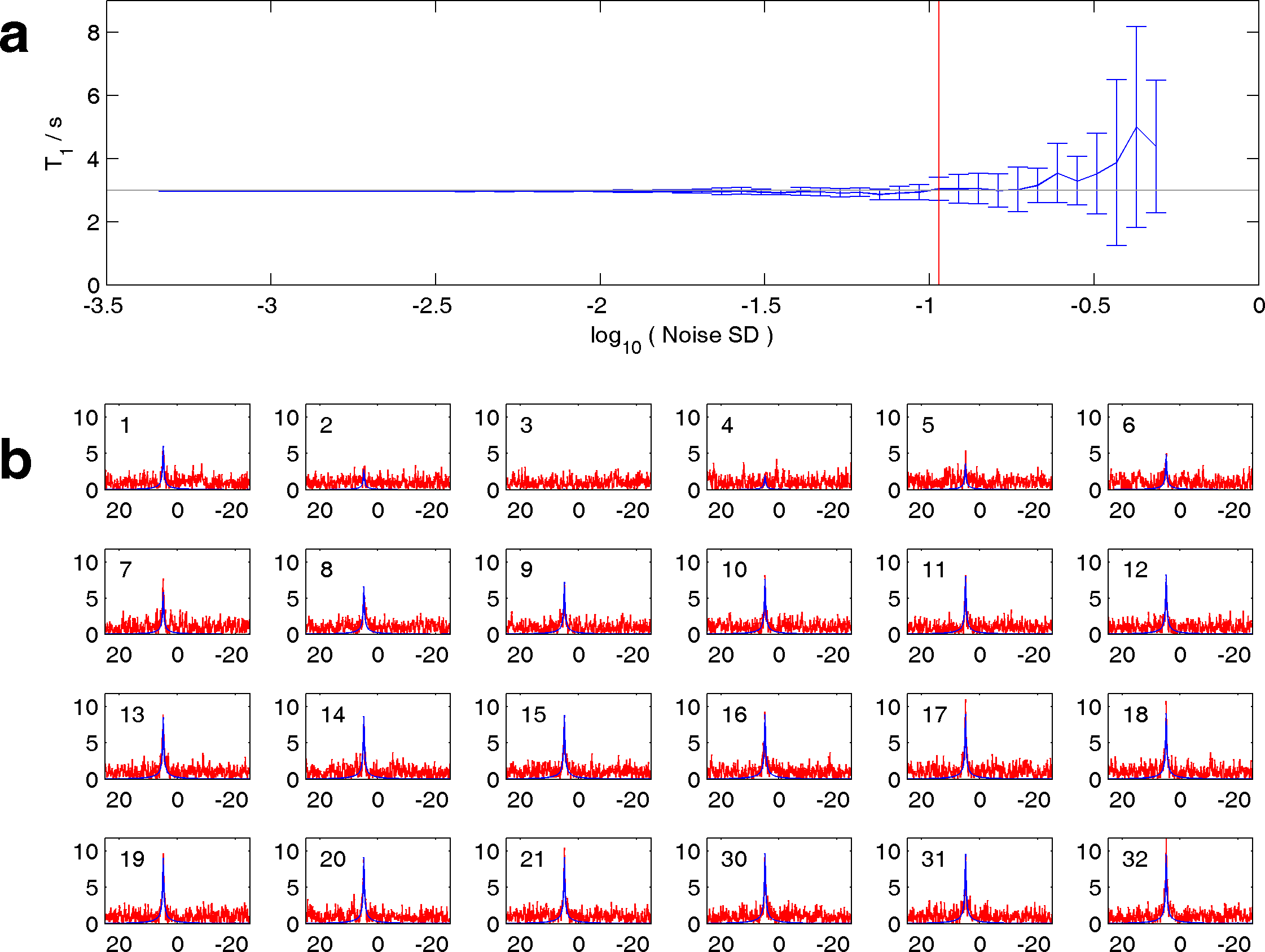


**Figure SI8**: Monte Carlo validation of the Look-Locker CSI fitting procedure. Simulated spectra were calculated by running the Bloch simulation model forward, setting PCr T_1_ = 3.0s, [PCr] = 1 and all other metabolite concentrations to zero. Randomly drawn noise with a certain standard deviation in the real and imaginary channels was added to give simulated spectra. These simulated spectra were then processed in the usual manner. (a) Mean ± SD for fitted T_1_ after 20 repetitions. (b) Montage showing simulated and fitted spectra for one repetition at the noise level denoted by the red vertical line in (a). This noise level is greater than in any of the experimental data, but the fitting performance is still reasonable.


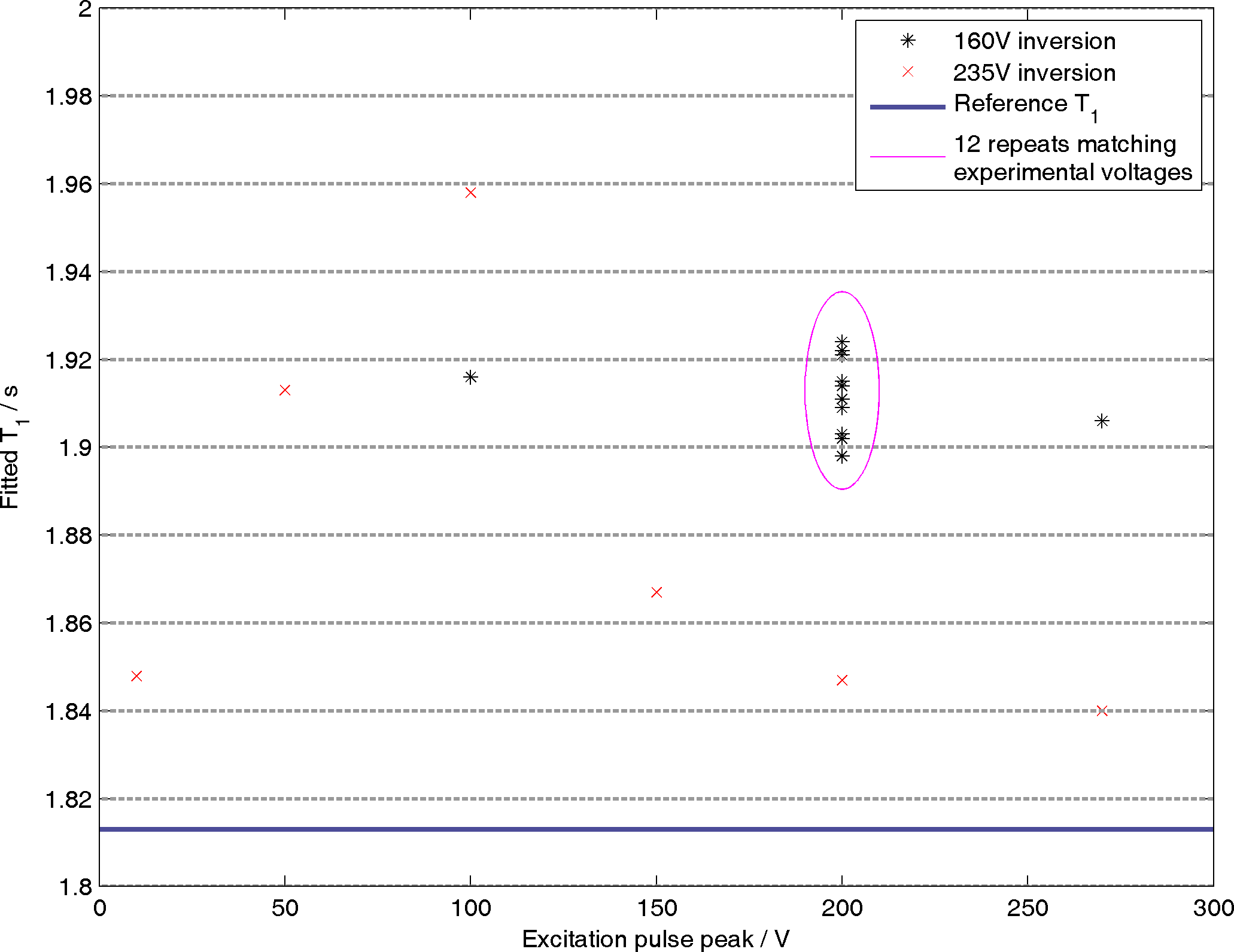


**Figure SI9**: Phantom validation of the Look-Locker CSI pulse sequence.
*Phantom:* The phantom shown in Figure SI1 was employed for a series of validation experiments.
*Reference T_1_:* A reference T_1_ = 1.813s (blue line) was recorded for KH_2_PO_4_ in the cube using non-localized inversion recovery with TR=15s, 14x TIs ranging between 50ms and 9000ms, and fitting to s(TI) = A - B exp(-TI/T_1_).
*Look-Locker CSI*: To simulate behaviour in vivo, the red KH_2_PO_4_ cube (see Figure SI1) was positioned 8cm from the RF coil where γB_1_ = 0.86 Hz V^-1^ (i.e. similar to that in the interventricular septum); the scanner reference frequency was set at +586Hz from the KH_2_PO_4_ peak; and the inversion pulses were centred at +774Hz relative to that. These frequency offsets match those for PCr in vivo. Other sequence parameters were matched with those used in vivo. In total, a series of 20x 38min Look-Locker CSI scans were performed, varying the excitation pulse voltages or inversion pulse voltages with each repeat.
*Analysis*: Data from the voxel containing the KH_2_PO_4_ cube were fitted to the Bloch equations in Matlab for each Look-Locker CSI scan. The fitting was started from a deliberately poor initial value T_1_^start^ = 4s. The fitted T_1_ from each 38min scan are plotted with an "x".
*Discussion*: All fitted T_1_s were within 0.15s of the reference T_1_ (i.e. <8% errors) in spite of the significant variation in voltages. This confirms that the in vivo experiment will be robust to deviations in B_1_. Finally, a series of repeated measurements (circled in magenta), all with voltages matching those in vivo (i.e. 160V inversion and 200V excitation) gave T_1_=1.913 ± 0.009s (mean ± SD). This is equivalent to an error of 5-6% relative to the reference value.

# Bloch simulation procedure

The Bloch simulation used for fitting Look-Locker CSI data involves the following principal stages:

1. Experimental parameters are read in from the DICOM headers.

2. The main parameter structure is initialised with default values.

3. The optimisation routine then begins. At each step it:

a) Updates the main parameter structure.

b) Calls the Bloch simulation code to compute a new set of model spectra as follows:

i). Vectors comprising the RF transmitted and the timing of spoiler gradients and of ADC events are prepared.

ii). For each multiplet component (i.e. 1x for PCr, Pi and the two 2,3-DPG singlets, 2x for α-ATP and γ-ATP doublets and 3x for β-ATP triplets), the T1, T2, Δν and the multiplet peak amplitude scaling factor are set using the main parameter structure.

ii). For each multiplet component, the M_x_, M_y_, and M_z_ time evolution is computed.

iii). The FIDs corresponding to each ADC event are extracted and returned to the optimisation routine.

c) Evaluates the residual norm, which expresses the goodness-of-fit.

d) Repeats steps a-c until a convergence criterion is reached.

4. The optimised parameters are stored and displayed on screen.
